# Strain-specific variation in the complement resistome of *Pseudomonas aeruginosa*

**DOI:** 10.1128/iai.00055-25

**Published:** 2025-08-11

**Authors:** Manon Janet-Maitre, Mylène Robert-Genthon, François Cretin, Sylvie Elsen, Ina Attrée

**Affiliations:** 1 University Grenoble Alpes, Institut de Biologie Structurale-IBS, UMR5075, Team Bacterial Pathogenesis and Cellular Responses 55543 , Grenoble, France; Universite de Geneve, Genève, Switzerland

**Keywords:** *Pseudomonas aeruginosa*, complement system, bloodstream infections, LPS, Tn-seq

## Abstract

Bloodstream infections caused by *Pseudomonas aeruginosa* are associated with high mortality rates. The complement system, a key component of the innate immune response, plays a major role in eliminating *P. aeruginosa* from human blood. However, the sensitivity of *P. aeruginosa* strains to plasma varies widely, ranging from highly sensitive to persistent or fully resistant. Although most studies use model strains, the species genomic and phenotypic diversities suggest more complex interactions with complement than previously appreciated. In this study, we characterized the plasma resistome of *P. aeruginosa* using Tn-seq in three strains with varying levels of plasma sensitivity. A gain-of-function screen in the plasma-sensitive strain PA14 revealed numerous bacterial factors influencing plasma resistance, including components of the RetS-LadS/Gac/Rsm regulatory pathway and outer membrane porins. In the plasma-resistant strains CHA and YIK, Tn-seq analysis indicated that each strain relies on a distinct, limited set of proteins to evade complement-mediated killing. Despite these differences, we identified common mechanisms across all three strains. These include the production of exopolysaccharides (EPSs), the presence of surface appendages, and modifications in the O-specific antigen. Notably, we identified Ssg and Crc as shared contributors to plasma resistance. Although deletion mutants lacking *ssg* and/or *crc* exhibited reduced survival in plasma, a subpopulation of these mutants was able to persist during prolonged exposure. Overall, this work provides new insights into the complex interplay between *P. aeruginosa* and the human complement system in the context of bloodstream infections and raises concerns regarding the efficacy of therapies that target individual virulence factors.

## INTRODUCTION

Bacterial bloodstream infections (BSIs) represent a huge burden for modern healthcare. BSIs caused by *Pseudomonas aeruginosa* exhibit the highest mortality rate (1, 2). This leading nosocomial pathogen causes various primary infections, such as burn wounds, pulmonary and urinary tract infections (3). Using its arsenal of virulence factors, it is able to breach both epithelial and endothelial barriers ([4–7], reviewed in [8]). Once in the bloodstream, *P. aeruginosa* faces the host immune response. In a previous study, we showed the complement system to be the main innate immune component responsible for the elimination of *P. aeruginosa* in the blood (9). The complement system is an enzymatic cascade of more than 30 proteins, whose activation results in the formation of the membrane attack complex (MAC) in the outer membrane of the target pathogen. Complement-mediated killing happens through three main steps including complement activation, assembly of the C5b-9 MAC on the outer membrane, and penetration of the MAC into the bilayer, eventually leading to bacterial lysis (10, 11). *P. aeruginosa* evolved strategies to evade those three steps. The secretion of proteases including AprA and LasB, cleaving C1, C2, and C3 or the secretion of ecotin, a protease inhibitor, leads to the blockade of complement activation (12–14). *P. aeruginosa* can also recruit host complement inhibitors to its surface, notably through the exposure of the elongation factor Tuf or the dihydrolipoamide dehydrogenase Lpd ([15, 16], reviewed in [8]). These immune evasion proteins can recruit the Factor H family of complement regulators, resulting in C3b degradation and preventing downstream proteolysis cascade. Finally, *P. aeruginosa* can modify its surface by producing exopolysaccharides (EPSs) such as alginate. When acylated, alginates decrease bacterial opsonization by the C3b molecule (17, 18).

Sensitivity of *P. aeruginosa* to the complement system varies widely. The survival rate of strains in human plasma/serum ranges from full resistance to high sensitivity and persistence and was not found to correlate with the serotype or toxin content (9). Some strains, although mostly sensitive to plasma-dependent killing, persist over a long incubation in plasma (9). Herein, the terms “resistance” and “persistence” will be used as defined in the context of antibiotics, with “resistance” attributed to a heritable resistance factor and “persistence” describing a situation where a tolerant subpopulation is able to survive, whereas the rest of the population is eliminated (19). A gain-of-function screen identified the first molecular factors involved in *P. aeruginosa* persistence in plasma. However, the screen was performed with the clinical strain IHMA879472 (IHMA87), a member of a distant clade of *P. aeruginosa*, recently reclassified as *Pseudomonas paraeruginosa* (20–22).

Several studies documented the important genetic and phenotypic diversity of bacterial strains within a *P. aeruginosa* species (22–24), with its pangenome accounting for more than 50,000 genes (22). Given the high intra-species genomic diversity and variability in plasma sensitivity, this work aims at characterizing strategies used by *P. aeruginosa* to evade complement killing in the blood, using genome-wide transposon mutagenesis (Tn-seq) on three strains belonging to different phylogenetic groups (9, 23–25). We found strain-specific sets of factors with only little overlap between strains. However, some common strategies used by *P. aeruginosa* to evade plasma killing could be drawn. We also report that targeting some factors may not eliminate resistant bacteria from human plasma but generate a persistent subpopulation. This work not only provides insight into a more global comprehension of *P. aeruginosa*-complement system interplay but also raises questions on the targeted antimicrobial or anti-virulence strategies developed on a unique bacterial strain.

## RESULTS

### Choice of strains and global Tn-seq analysis

**Fig 1:**
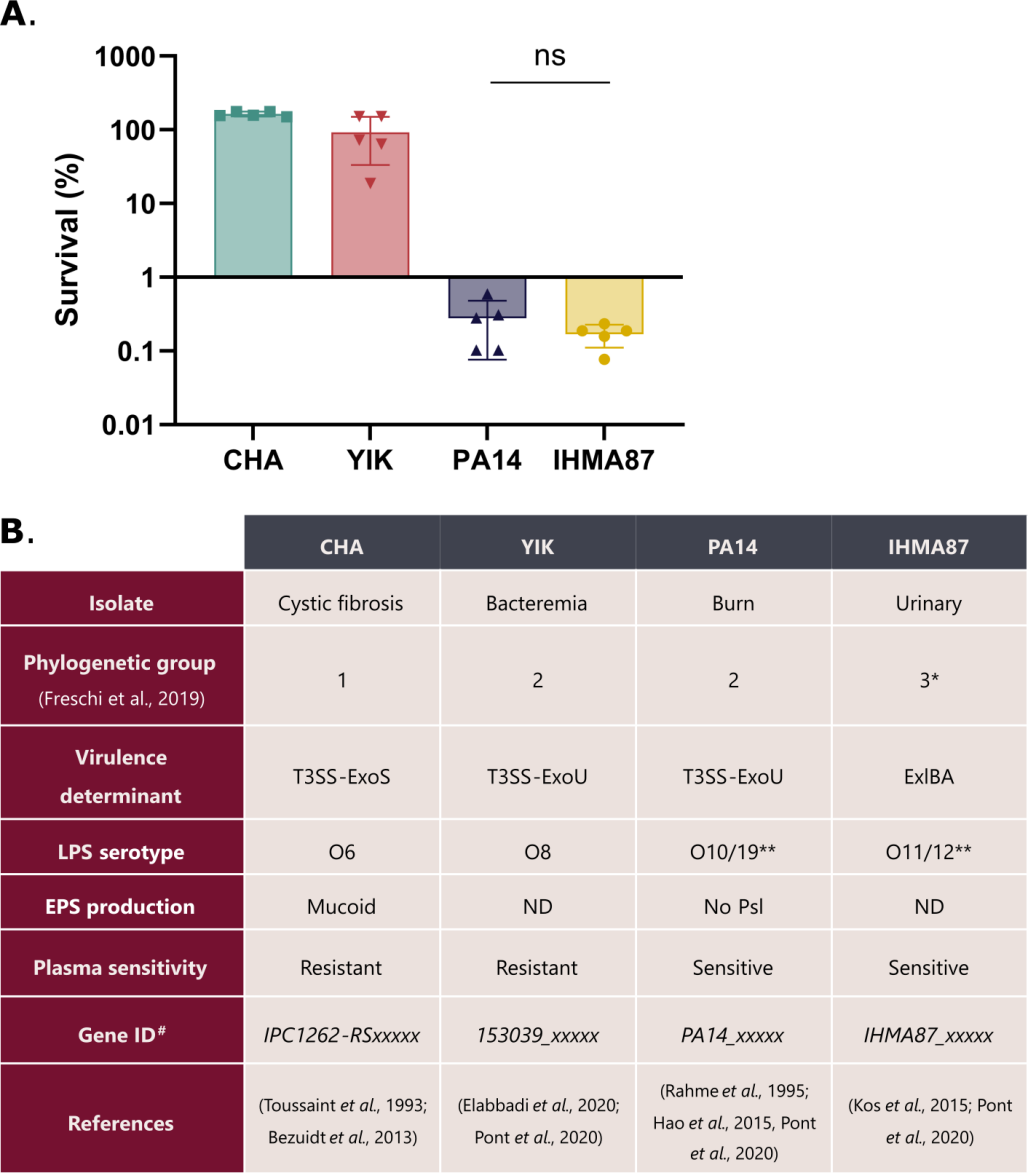
*P. aeruginosa* strains show variable survival in plasma. (**A**) Bacterial survival after a 3 h incubation in human pooled plasma was calculated relative to T0 using colony-forming units (CFU) measurements. Data were log-transformed for one-way ANOVA statistical analysis followed by Tukey’s test. All differences in survival between strains were found to be statistically significant except between IHMA87 and PA14. ns: non-significant. (**B**). Characteristics and origin of strains used in this study. ND (not determined).*: Recently reclassified as *P. paraeruginosa.* **: Reactivity against both serotype antibodies (9, 32). ^#^: Gene ID found in supplemental tables.

The three strains used in this work belong to the two major clades of *P. aeruginosa* and have different characteristics in terms of plasma sensitivity, serotype, and toxin content (Fig. 1). The strains CHA, a hyper producer of the EPS alginate, isolated from the lungs of a cystic fibrosis patient (26, 27), and YIK, a recent bacteremia isolate (28), are both resistant to human plasma (~100% and ~91% survival, respectively, Fig. 1A). On the contrary, the laboratory strain PA14 (29) is highly sensitive to plasma and forms a persistent subpopulation, similarly to IHMA87 used in a previous study (Fig. 1A, [9, 30, 31]). PA14, CHA, and YIK harbor the type III secretion system (T3SS) with different toxin contents (Fig. 1B). IHMA87 synthesizes the ExlBA two-partner secretion system instead of the T3SS and is part of the third, the most distant phylogenetic group of *P. aeruginosa* (22, 23)*,* recently reclassified as *P. paraeruginosa* (20).

We first generated transposon libraries in the three described strains, each containing above 300,000 mutants using the Himar-1 transposon carried by pBTK24. Between 74% and 91% of *P. aeruginosa* genes contained transposon insertions (between 5821-6125 genes in the genome), consistent with previous studies reporting an average of 400–800 essential genes per strain (Table S1, [33, 34]). The screen on IHMA87 (21) was done in parallel, as a comparison for PA14. Transposon mutant libraries were challenged for 3 h in 90% pooled plasma from healthy donors (output) or heat-inactivated plasma (HIP), unable to kill *P. aeruginosa,* as a control (input). Survivor cells were then recovered in a rich medium prior to genomic DNA extraction, bank preparation, and Illumina-based sequencing. The screening setup enables the identification of mutants with altered survival phenotypes by measuring changes in mutants’ abundance across the genome, that is, decreased survival for plasma-resistant strains (CHA and YIK) or enhanced survival for plasma-sensitive strains (PA14). As expected, the overall survival of the pool of PA14 transposon mutants was about 3 logs higher than that of the parental strain, as already noted for the sensitive strain IHMA87 (Fig. S1, [21]).

**Fig 2:**
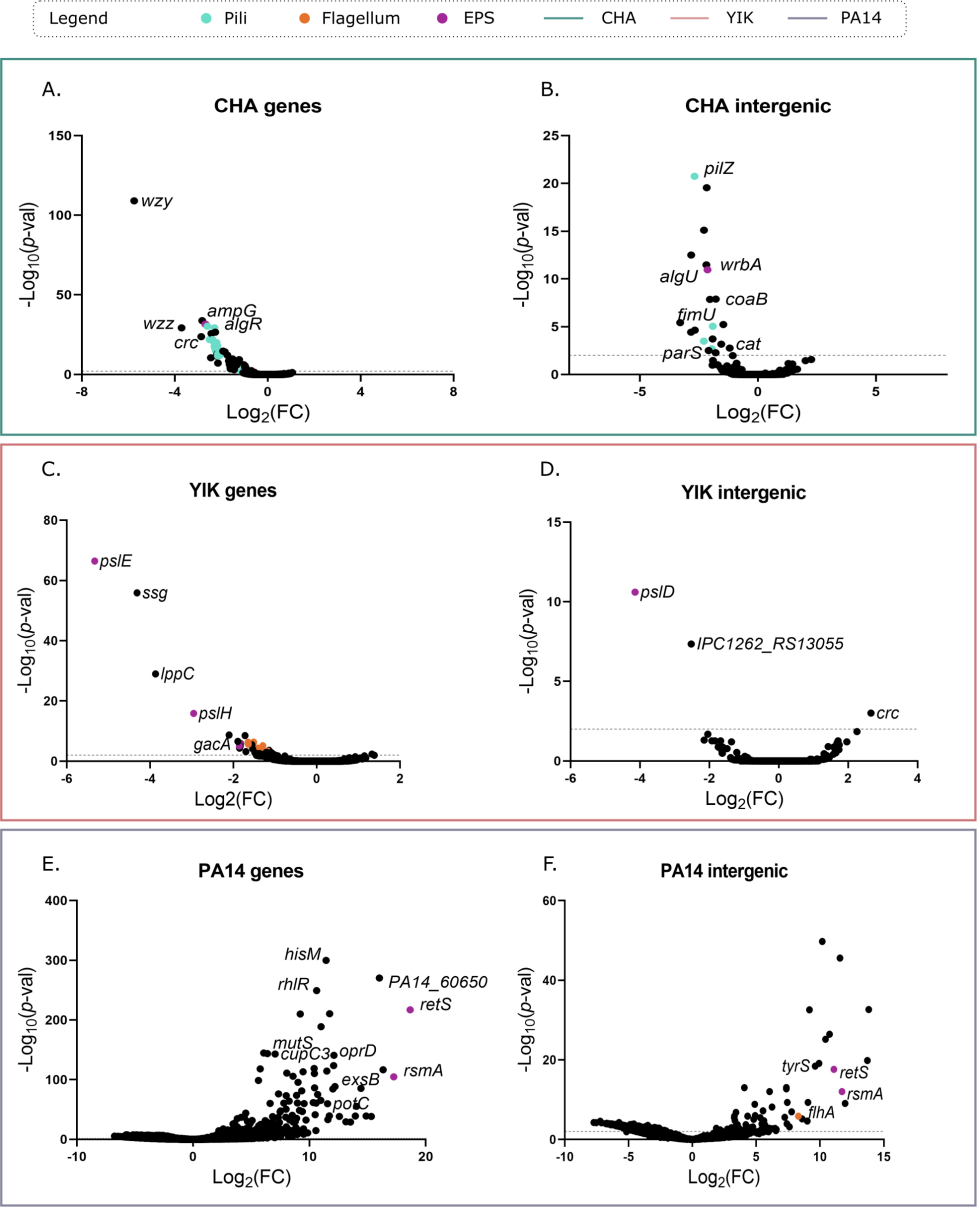
Bioinformatic analysis of Tn-seq data both in genes and intergenic regions. Volcano plot representation of the data obtained for CHA (**A, B**), YIK (**C, D**), and PA14 (**E, F**) both in genes (**A, C, E**) and intergenic regions (**B, D, F**). Sequencing data were analyzed using the Galaxy platform (35) and visualized using GraphPad Prism 9. Pathways are highlighted in different colors. Dotted line represents a *P_val_* = 0.01.

Bioinformatics analysis of the sequencing reads was performed for both coding and intergenic regions due to the possible polar effect of the inserted transposon. Depending on the orientation and insertion site of the transposon, which has an outward-directed *P_tac_* promoter, the insertion can either inactivate the target gene or overexpress the neighboring gene(s), as shown in (33). The analysis of the Tn-seq data showed that plasma resistance of CHA and YIK relies on a few determinants as the screen identified 45 and 37 hits, respectively, using Log_2_(fold change (FC))>1 and adjusted *P-*val (*P*_adj_) <0.01 as thresholds (Fig. 2A through D; Tables S2, S3, S4 and S5).

Conversely, more than 600 hits were found to improve PA14 survival in plasma (682 hits with Log_2_(FC) >1, *P*_adj_ <0.01, Tables S6 to S8). As observed in the volcano plot Fig. 2E and F, few transposon insertions negatively affected PA14 survival in human plasma given the already high sensitivity of the parental PA14 strain to complement-dependent killing. Members of the RetS-LadS/Gac/Rsm regulatory system, involved in *P. aeruginosa* phenotypic switch between planktonic and biofilm lifestyles, had improved plasma resistance including *retS* and *rsmA* with Log_2_(FC) of 18.7 and 17.2, respectively (Table S6, [36, 37]). Transposon insertion disrupting the gene of several outer membrane porins, for example, *oprD* and *oprP*, also drastically improved PA14 resistance to plasma with Log_2_(FC) of 12.1 and 8.0, respectively. The role of OprF, another outer membrane porin, as a C3 binding acceptor molecule has been previously reported (38). This result suggests that other outer membrane porins may also serve as C3 acceptors. Additionally, disruption of genes involved in metabolisms of nucleotide (pyrimidines: *pyrC, pyrE,* and purines: *purH*), amino acids (e.g., *glyS, hisM*), energy production (*sucC*), biotin biosynthesis (*bioA*), etc., led to increased plasma resistance, in line with the data from the strains IHMA87 (21).

Surprisingly, no hit was shared between the two plasma-resistant strains CHA and YIK (Fig. S2), although the presence and the synteny of most genes were conserved at the genomic level. When comparing the plasma-sensitive strain PA14 with the data obtained for IHMA87, 99 common genes showed Log_2_(FC) >1, *P*_adj_ <0.01 (Fig. S2 and [21]). However, except for *retS*, which was one of the top hits for both PA14 and IHMA87 (Tables S6 to S8), the other hits had highly variable Log_2_(FC) between the two strains, suggesting that their contribution to protection against the plasma-dependent killing is variable among the strains. The limited redundancy found between the different strains is coherent with what was observed in a similar study on *Klebsiella pneumonia* (39). Although the individual gene sets were not strictly shared between the strains tested, we identified common strategies used to evade complement-mediated killing across strains.

### Contribution of EPS to survival in plasma is strain-dependent

*P. aeruginosa* can produce three types of EPS: Psl, Pel, and alginate. Both Psl and alginates were previously described to protect *P. aeruginosa* from opsonization by C3b ([17, 18, 40], reviewed in [8]). Here, we confirm EPSs, and particularly Psl, to have a central role in *P. aeruginosa* survival in plasma, but in a strain-dependent manner.

**Fig 3:**
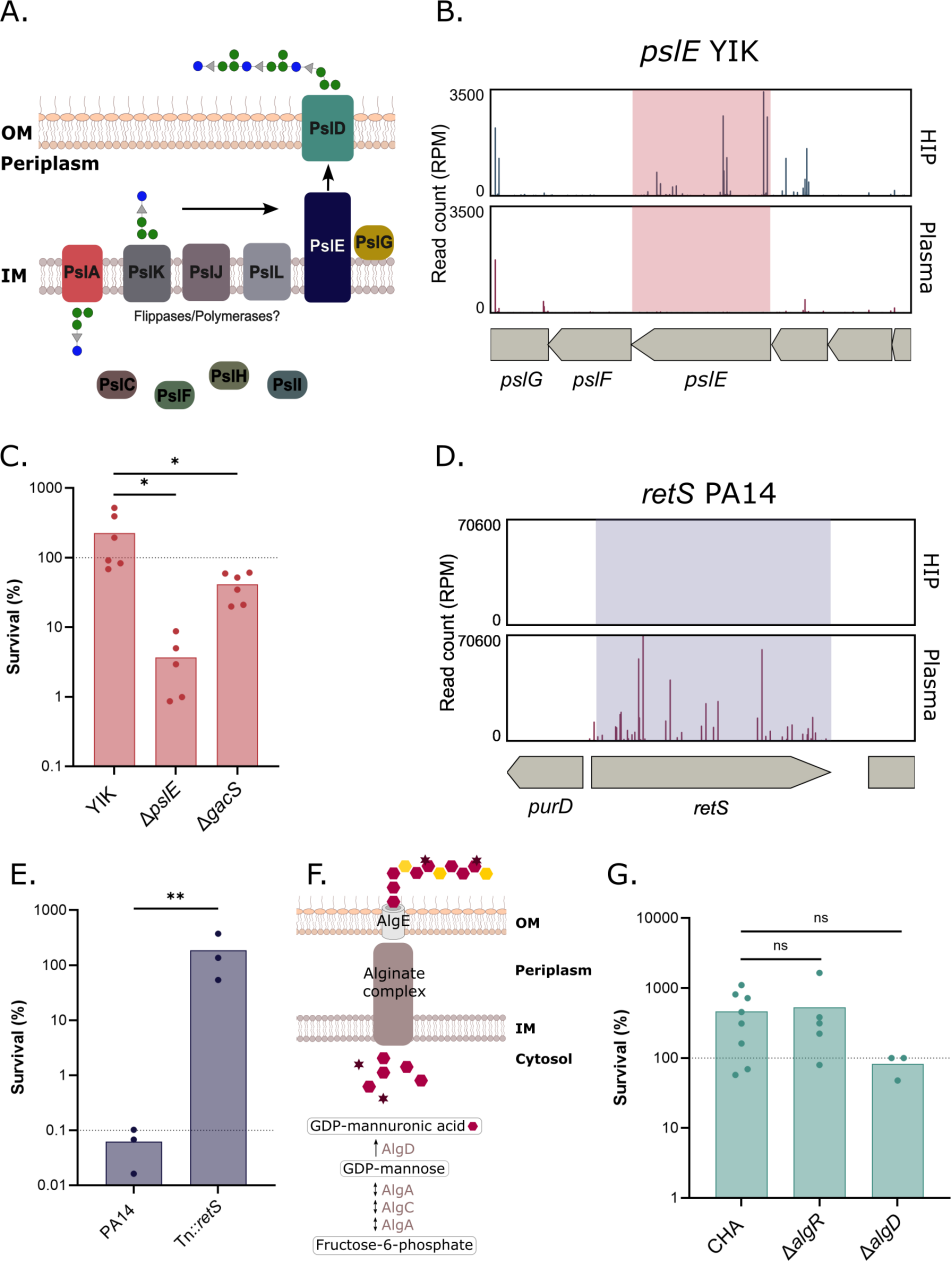
Exopolysaccharides contribute to survival in plasma in a strain-dependent manner. (**A**) Scheme of Psl production and export in *P. aeruginosa*. (**B**) Tn-seq profile of *pslE* genomic environment in YIK showing the number of normalized reads in input (HIP) and output (plasma). (**C**) Bacterial survival after a 3 h incubation in human pooled plasma of YIK, YIK*∆pslE, and* YIK∆*gacS*. (**D**) Tn-seq profile of *retS* genomic environment in PA14, showing the number of normalized reads in input (HIP) and output (plasma). (**E**) Bacterial survival after a 3 h incubation in human pooled plasma of PA14 and PA14Tn::*retS*. (**F**) Schematic of alginate synthesis and export in *P. aeruginosa*. (**G**) Bacterial survival after a 3 h incubation in human pooled plasma of CHA and CHA*∆algR* and CHA∆*algD*. Note that the survival of CHA *∆algD* is ~100%. A log transformation was performed on the data set and a one-way ANOVA, followed by Tukey’s test. The dashed line represents the expected survival for the strain shown. ns: non-significant, *: *p_val_* <0.05,**: *p_val_* <0.01.

Psl is a neutral exopolysaccharide, which is important in *P. aeruginosa* attachment and maintenance of the biofilm architecture. Although Psl biosynthetic genes are encoded in the same locus, the detailed role of individual genes in Psl synthesis is still unclear (41). Briefly, Psl is thought to be produced by the Psl biosynthesis machinery. PslA is proposed to assemble oligosaccharide repeating units that are then polymerized by PslE. PslG controls the size of the oligosaccharide. Finally, Psl is exported to the bacterial surface through the transporter PslD (Fig. 3A; [41, 42]). Psl was shown to reduce bacterial opsonization including reduced C3b, C5, and C7 binding at the bacterial surface (40). However, the role of Psl in MAC-dependent bacterial killing is still under debate. Jones and Wozniak (43) showed a slight beneficial effect of Psl on the survival of *P. aeruginosa* mucoid strains to serum killing, which was not observed by Mishra et al. (40).

In the non-mucoid, plasma-resistant strain YIK, transposon insertions in three genes within the *psl* locus drastically reduced bacterial survival in plasma (*pslE* – Log_2_(FC) = −5.33, *pslH* – Log_2_(FC) = −2.95, *pslD* – Log_2_(FC) = −1.86, (Fig. 2C, D and 3B; Tables S4 and S5). We engineered a *pslE* deletion mutant (Δ*pslE*) in YIK and assessed its survival rate in human plasma. On average, 20 times fewer bacteria were recovered after the exposure of YIKΔ*pslE* to plasma compared with the parental strain, supporting the role of Psl in complement evasion in YIK (Fig. 3C).

Furthermore, the RetS-LadS/Gac/Rsm regulatory system, which participates in the phenotypic switch between planktonic and biofilm growth and regulates the expression of EPS loci (37, 44), was found as a hit in several strains. For example, in YIK, the deletion of *gacS* led to a 2-fold decrease in bacterial counts compared with the parental strain when challenged with plasma (Fig. 3C). Similarly, in the plasma-sensitive strain PA14, a transposon insertion mutant in the gene *retS,* isolated directly as the screen output (PA14::Tn*retS*), displayed a 1,000-fold increased survival (Log_2_(FC) = 18.66) (Fig. 2E, F, 3D and E), in line with our results in the strain IHMA87 (Log_2_(FC) = 9.11, [21]). The PA14 strain is a natural mutant in *ladS* and lacks several *psl* genes (45, 46). In addition, alginate is not typically produced by PA14 under standard conditions (47). Therefore, although RetS has a pleiotropic role, we hypothesize that the Tn insertion in *retS* probably activates the synthesis of Pel EPS, thereby improving PA14 survival in plasma.

Surprisingly, transposon insertion mutants in either Pel, Psl*,* or alginate biosynthetic pathways in hyper-mucoid strain CHA showed unaltered plasma resistance (Fig. S3; Tables S2 and S3). Accordingly, a nonmucoid *algD* mutant, in which the last step of the alginate biosynthetic pathway is disrupted (Fig. 3F), displayed comparable plasma resistance to the parental strains (Fig. 3G). Previous studies reported that alginates and especially acetylated alginates decreased C3b-mediated bacterial opsonization and therefore increased bacterial survival in clinical strains isolated from cystic fibrosis patients (17, 18), which was not confirmed in our work using the alginate hyper-producer strain CHA. It is worth noting that transposon-insertion mutants in *algU* or *algR* showed decreased abundance following plasma challenge (Tables S2 and S3). Both genes, *algU* encoding the alternative sigma factor and *algR* encoding for a global regulator, are key players in *P. aeruginosa* virulence (48–51). However, the resistance to plasma of an *algR* deletion mutant was found unaffected when tested individually, as opposed to tested in-pool during the Tn-seq experiment (Fig. 3G). Due to the large size of AlgU and AlgR regulons involved in the response to envelope stress and virulence (49, 51), both proteins may affect CHA plasma resistance in an alginate-independent manner. Hence, although alginates can increase plasma resistance in sensitive strains (e.g*.* IHMA87 [21]), they are not crucial EPSs for evasion in other strains.

Together, our work shows that EPSs, mostly Pel and Psl, provide potent but strain-dependent protection against the complement system in plasma.

### Surface appendages favor survival in plasma

**Fig 4:**
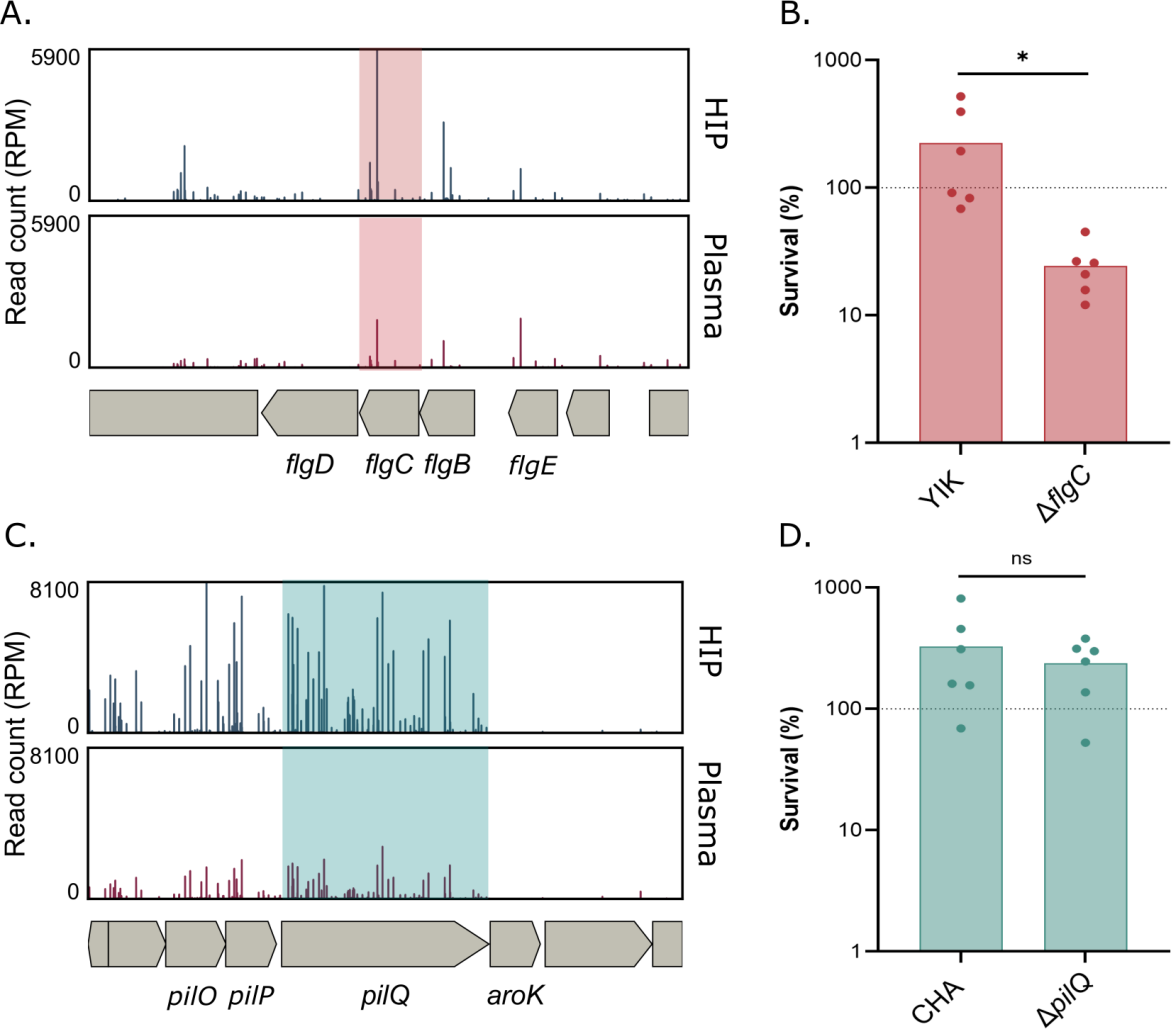
The strain-specific role of surface appendages in survival in plasma. (**A**) Tn-seq profile of the *flgC* genomic environment in YIK showing a number of normalized reads in input (HIP) and output (plasma). (**B**) Bacterial survival after 3 h incubation in human pooled plasma of YIK and YIK*∆flgC*. (**C**) Tn-seq profile of *pilQ* genomic environment in CHA showing number of normalized reads in input (HIP) and output (plasma). (**D**) Bacterial survival after a 3 h incubation in human pooled plasma of CHA and CHA∆*pilQ*. The dashed line represents the expected survival for the strain shown. The data were log-transformed, and a *t*-test was performed. ns: non-significant, *: *P_val_* <0.05.

Bacterial flagella and pili are potent immune activators (52). In this work, we found that the type IV pili (T4P) and the flagellum are surface components providing an advantage to strains CHA and YIK in plasma (Fig. 4; Tables S2 to S5). In the screen with the strain CHA, the transposon mutants in 18 T4P-related genes had a reduced abundance in plasma compared with the inactivated plasma (Tables S2 and S3). In addition, in the strain YIK, Tn mutants were underrepresented for 12 flagella-related genes (Tables S4 and S5).

To validate the data, we created a *flgC* deletion mutant in YIK and a *pilQ* deletion mutant in CHA and challenged them individually in human plasma. In accordance with Tn-seq sequencing reads (Fig. 4A), the deletion in *flgC,* encoding a component of the flagellar basal body (53), resulted in a 4-fold drop in survival of YIK (Fig. 4B). Surprisingly, the deletion in CHA of a gene essential for pili synthesis, *pilQ* (54, 55), did not confirm the Tn-seq data (Fig. 4C and D). However, considering the Log_2_(FC) and *P*-values of several distinct T4P-related hits in Tn-seq data sets, it is unlikely that pili genes were false positives. One hypothesis could be that the identified transposon mutants had a competitive disadvantage in the pooled Tn-seq screen compared with the challenge of an individual deletion mutant.

Several studies point toward a protective role of flagella and pili against serum-mediated killing. In *Streptococcus pyogenes*, pili were shown to bind haptoglobin, an immunosuppressive serum protein, and sequestration provided a survival advantage in serum (56). Tad pili, one type of T4P in *Vibrio vulnificus,* protect bacteria from complement-mediated killing in serum (57). In addition to pili, flagellar components were also found to be required for serum resistance, such as FliG in *Escherichia coli* (58).

Altogether, the flagellum contributes to YIK resistance to human plasma, potentially by binding serum components, such as opsonins or other inhibitors, thereby reducing complement-mediated lysis of *P. aeruginosa*.

### *ssg* and *crc* mutants persist in the plasma

Previous studies showed that MAC insertion in the outer membrane is impacted by the length of O-specific antigens (OSA - [59]). *P. aeruginosa* LPS is synthesized in two forms, either bound with a D-Rhamnose homopolymer known as Common Polysaccharide Antigen (CPA) or OSA, a heteropolymer of sugar repeats with repeat composition determined by the *wbp*_OSA_ locus (60, 61).

**Fig 5:**
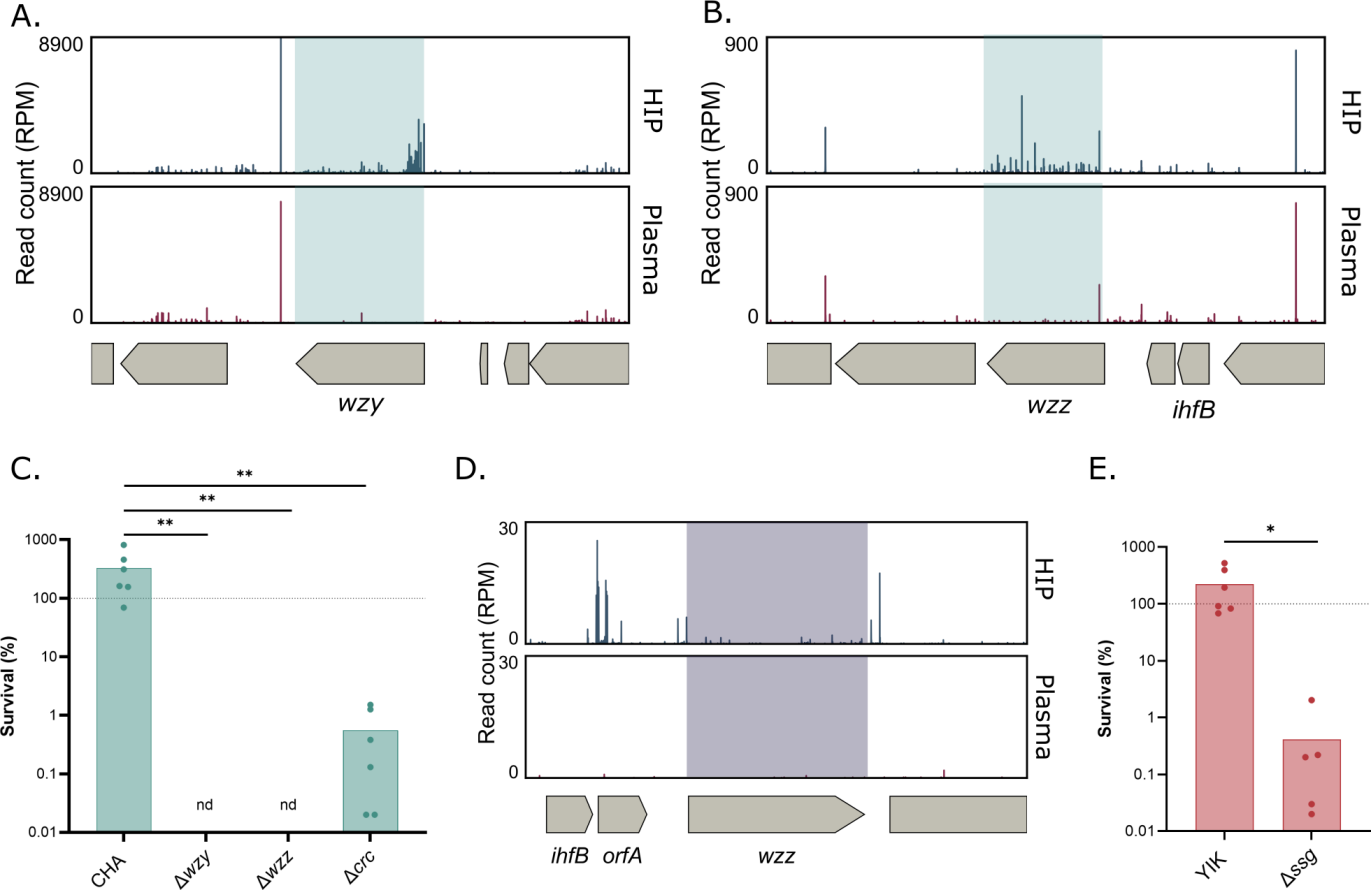
LPS is crucial for survival in plasma. Tn-seq profile of *wzy* and *wzz* genomic environment in CHA (**A and B**) showing a number of normalized reads in input (HIP) and output (plasma). (**C**) Bacterial survival after a 3 h incubation in human pooled plasma of CHA, CHA∆*wzy,* CHA∆*wzz, and* CHA∆*crc*. nd: non detected. Data were log-transformed, and a one-way ANOVA and Tukey’s test were performed. (**D**) Tn-seq profile of *wzz* genomic environment in PA14 in input (HIP) and output (plasma). (**E**) Survival after a 3 h incubation in human pooled plasma of YIK and YIK∆*ssg*. Data were log-transformed, and a *t*-test was performed. *: *P_val_* <0.05.**: *P_val_* <0.01. The dashed line represents the expected survival for the strain shown.

The length of newly generated OSA is regulated by Wzz1 and 2, before its attachment to the nascent lipid A (59, 62, 63). In the mucoid and plasma-resistant strain CHA, transposon insertions in *wzy* and *wzz* represented the two best hits and led to elimination of mutants from plasma (Log_2_(FC) = −5.75 and Log_2_(FC) = −3.71, respectively) (Tables S2 and S3; Fig. 5A and B), which was confirmed by the complete elimination of clean deletion mutants of either *wzy* or *wzz* in CHA (Fig. 5C). Similarly, a transposon insertion in the promoter of *wzz,* probably leading to the overexpression of the *wzz* gene, was enriched in plasma in the sensitive strain IHMA87 (21). Although *wzz* was not a significant hit in the PA14 Tn-seq data set, due to the high sensitivity of PA14 to human plasma, the zoomed Tn-seq profile suggests a similar role of *wzz* in PA14 plasma resistance (Fig. 5D). Altogether, our data are in agreement with the literature.

In addition to *wzz* and *wzy*, two other genes with a potential role in LPS synthesis were identified in the screen: *ssg* and *crc*. The inactivation of *ssg*, a conserved gene encoding a putative glycosyltransferase, in the YIK strain led to a drastic survival drop (Log_2_(FC) = −4.31, Fig. 5E; Tables S4 and S5), whereas *crc,* with a Log_2_(FC) = −2.86 was the second top hit in CHA (Fig. 5C; Tables S2 and S3).

**Fig 6:**
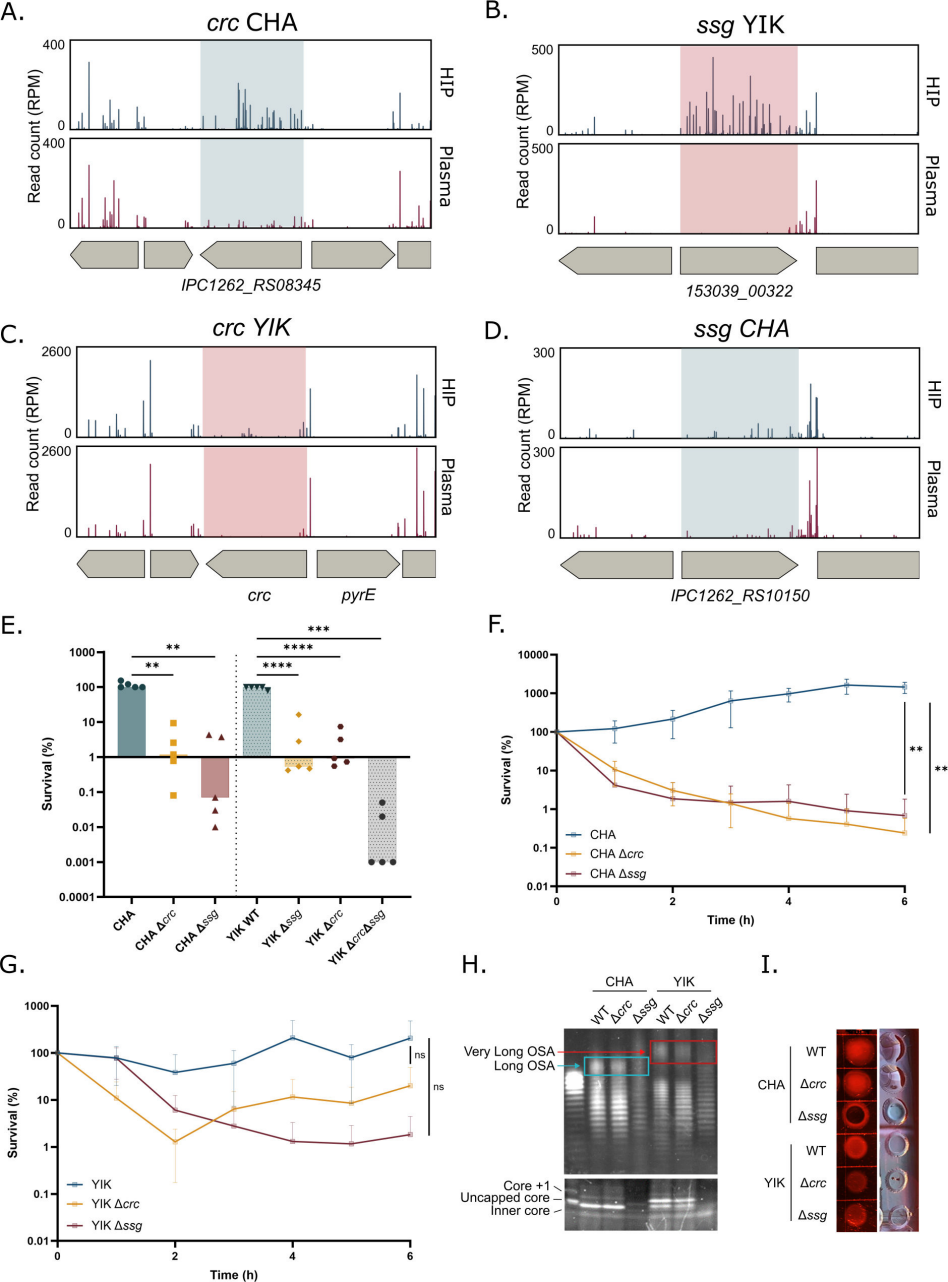
*crc* and *ssg* mutants persist in plasma. (**A**) Tn-seq profile of *crc* (*IPC1262_RS08345*) genomic environment in CHA showing number of normalized reads in input (HIP) and output (plasma). (**B**) Tn-seq profile of *ssg* (*153039_00322*) genomic environment in YIK showing the number of normalized reads in input (HIP) and output (plasma). (**C**) Tn-seq profile of *crc* genomic environment in YIK and (**D**) *ssg* genomic environment in CHA. (**E**) Bacterial survival after a 3 h incubation in human pooled plasma of CHA, CHA*∆ssg,* and CHA∆*crc and* YIK*,* YIK∆*ssg,* YIK∆*crc,* and YIK∆*crc∆ssg*. Data were log-transformed for statistical analysis by a one-way ANOVA followed by a Welch and Brown-Forsythe test. (**F**) Survival kinetics of CHA, CHA∆*ssg,* and CHA∆*crc* over a 6-h incubation in human pooled plasma. (**G**) Survival kinetics of YIK, YIK∆*ssg, and* YIK∆*crc* over a 6-h incubation in human pooled plasma. (**F, G**) AUC of each biological replicate was calculated, and a one-way ANOVA was applied followed by Tukey’s test. *: *P_val_* <0.05, **: *P_val_* <0.01, ***: *P_val_* <0.001, ****: *P_val_* <0.0001. ns: non-significant. (**H**). LPS extraction and silver stain of CHA, CHA∆*ssg,* and CHA∆*crc* and YIK, YIK∆*ssg,* and YIK∆*crc*.The blue box shows long O-specific antigens (OSA) in CHA and CHA*∆crc,* which are absent in CHA*∆ssg*. The red box shows very long OSA in YIK and YIK*∆crc,* absent from YIK*∆ssg*. (**I**) Congo red binding of colonies of WT, ∆*ssg,* and ∆*crc* in CHA and YIK genetic backgrounds.

A *crc* deletion mutant tested individually showed a drop in plasma resistance, reaching less than 1% survival after a 3 h challenge compared with the fully resistant parental strain. In the Tn-seq data, *crc* was neither found as a significant hit in the YIK strain nor was *ssg* in CHA. Therefore, we re-examined those genes in the original Tn-seq data sets. Although the data were not significant due to fewer insertion mutants in the input library, a similar trend could be observed in both strains (Fig. 6A through D).

We therefore engineered *ssg* and *crc* deletions in both strains. The four mutants displayed survival rates between 1.6% and 4.1% (Fig. 6E). Surprisingly, although deletions in *wzz* and *wzy* led to full bacterial elimination, *crc* and *ssg* mutants became more sensitive but were not fully eliminated after a 3 h challenge in plasma (Fig. 6E). We therefore investigated the killing kinetics of *ssg* and *crc* mutants. The killing curves obtained by a challenge of the different mutants in plasma for up to 6 h were reminiscent of those obtained for persistent strains, PA14 or IHMA87 (9), with a rapid decrease in survival within initial 2 h and then a stabilization to the levels of 1%–10% (Fig. 6F and G). The plasma sensitivity after 3 h of a double mutant in YIK was lower than that of either single mutant individually, suggesting distinct roles of *ssg* and *crc* in plasma survival (Fig. 6E).

### Characterization of *ssg* and *crc* mutants for LPS and EPS

Literature mining suggested a possible link between *ssg* and *crc,* OSA, and EPS. A *ssg* mutant in *Pseudomonas alkylphenolia* was found to lack OSA and displayed altered composition of EPS lacking fucose and mannose (64). In *P. aeruginosa*, the *ssg* mutant in the PAO1 strain was impaired in auto-aggregation due to its LPS hydrophobic profile and loss of OSA, whereas in PA14, a *ssg* mutant displayed increased susceptibility to polymyxin B and increased permeability (65–67). *crc* encodes a post-transcriptional global regulator of carbon metabolism (68). Among other targets, Crc regulates genes involved in LPS biosynthesis (68). A *crc* deletion mutant in PAO1 had an increased proportion of LPS molecules with longer OSA compared with the parental strain.

Given the known importance of LPS in bacterial sensitivity to plasma, we first analyzed LPS content of Δ*crc* and Δ*ssg* mutants in CHA and YIK genetic backgrounds. The LPS quantity and pattern were indistinguishable between Δ*crc* mutants and their respective parental strains (Fig. 6H), in contrast with the previous report (68). LPS extraction and staining do not enable clear discrimination between high molecular weight OSA and CPA. However, in agreement with the literature, fewer bands were observed in Δ*ssg* compared with both the WT and the Δ*crc* strains, suggesting the presence of CPA but the absence of OSA in Δ*ssg* (Fig. 6H, [65–67]). Additionally, the Δ*ssg* mutants lacked long OSA in CHA (Fig. 6H, blue box) and very long OSA in YIK (Fig. 6H, red box) and exhibited decreased amounts and shifts in the free LPS core, particularly striking in the CHA genetic background (Fig. 6H). In plasma challenge experiments, Δ*ssg* formed persisters, in contrast to Δ*wzz and* Δ*wzy* mutants (Fig. 6F and G, reviewed in [8]).

We then investigated the role of *crc* and *ssg* in EPS production using Congo red plates. CHAΔ*crc* and YIKΔ*crc* had apparent Psl production similar to that of the parental strain (Fig. 6I). Interestingly, the colony of CHAΔ*ssg* mutant was dark red and not shiny, indicating increased Psl production and reduced alginate production (69).

We conclude that although *crc* and *ssg* appeared as good targets in two resistant strains, their deletion led to persistence without complete bacterial elimination.

## DISCUSSION

In this work, we used Tn-seq in three genetically and phenotypically diverse strains to investigate the convergence of molecular mechanisms interfering with the complement system cascade and subsequent bacterial killing. Data analysis revealed no universally shared factors involved in plasma resistance across strains, consistent with previous findings in *K. pneumoniae* strains exposed to serum (39). However, we could identify common strategies used by the different strains. In addition, the results confirmed for one strain may be useful to understand plasma resistance in other strains as we highlighted for *crc* and *ssg* phenotypic analysis.

**Fig 7:**
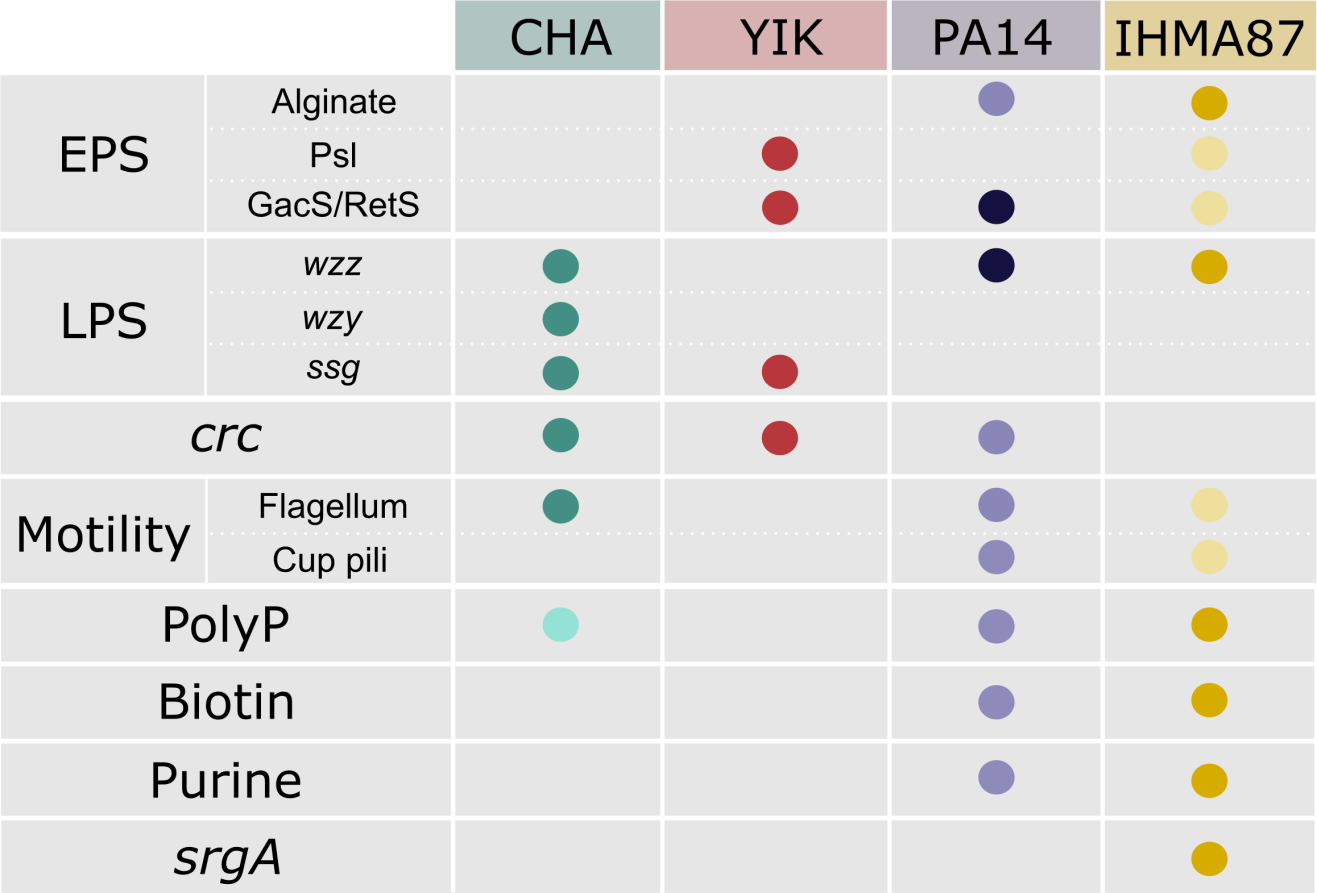
The plasma resistance of each *P. aeruginosa* strain depends on a unique combination of molecular factors. Dots represent the implication of the corresponding gene or pathways in resistance to plasma in the indicated strain either confirmed experimentally on individual mutants (darker color) or in Tn-seq data (lighter dots for CHA, PA14, and IHMA87) in this work or in (21).

*P. aeruginosa* genomic complexity has been highlighted by various studies (22, 23) serving as resources to adapt to different environments and stresses. In addition to the lack of conservation of some potential targets among strains, each strain has its own expression profile of virulence factors, which could also account for part of the diversity in molecular determinants for plasma resistance. In plasma-persistent strains, PA14 and IHMA87, the number of transposon insertions positively affecting global survival was more important, compared with the number of hits negatively affecting the resistance of CHA and YIK. Our results suggest a highly strain-specific defense mechanism against the complement system, involving a myriad of bacterial effectors that constitute a specific cocktail of bacterial factors, shaping a strain’s resilience to complement-mediated killing (Fig. 7).

Poulsen and colleagues investigated the core essential genome of nine strains of *P. aeruginosa* in five infection-relevant media including heat-inactivated serum, sputum, and urine (34). They propose that four strains are sufficient to establish a reliable essential genome. In this work, the variability was such that four strains were insufficient to assess the full complexity of the host-pathogen interface in the plasma of *P. aeruginosa*. This underscores the limitations of drawing broad conclusions from single-strain studies in host-pathogen interaction research. Complement system-mediated killing is initiated onto the bacterial surface and results in the formation of a pore in the outer membrane, eventually leading to bacterial lysis and death. As suggested in *Borrelia burgdorferi*, the presence of various outer membrane proteins was responsible for bacterial resistance to MAC-dependent killing (70). This was also evidenced in *K. pneumoniae*, in which the deletion of the gene coding for the outer membrane lipoprotein LppA led to an increased bacterial susceptibility to complement-mediated killing (71). Although the exact subsequent mechanism is still under debate, two main hypotheses are drawn. The first would imply that the formation of the MAC pore on the outer membrane allows the passage of C9, subsequently forming pores in the inner membrane and causing bacterial killing. The other possible molecular mechanism implies that the formation of pores in the outer membrane destabilizes the envelope, leading to the loss of envelope integrity and subsequent bacterial death. Both hypotheses were discussed in reference (72). The main resistance mechanism involves the inhibition of opsonization on the bacterial envelope and degradation of complement components (71). To reduce opsonization, bacteria can secrete EPS or modify their outer membrane composition to reduce the number of opsonin acceptors such as OprF (38). For this reason, we expected an enrichment of outer membrane proteins as the top hits of the screen. Surprisingly, the outer membrane proteins accounted for only 9.3%, 0%, 2.9%, and 3.2% for CHA, YIK, IHMA87, and PA14, respectively (Fig. S4). Conversely, all the strains had a high proportion of inner membrane-associated genes in the list of significant hits of about 25%–30%. These results are in agreement with the work of Heesterbeek et al. (72), which suggests the permeabilization of the inner membrane to be the rate-limiting step in MAC-dependent killing. In addition, the screen reports many cytoplasmic hits, which may exert an indirect function onto the bacterial envelope or metabolism to help the bacterium cope with the complement system-derived envelope stress. Global strategies, highlighted in other species such as *K. pneumoniae* or *Acinetobacter baumannii* (39, 73), were shared across strains including the production of protective exopolysaccharides and the synthesis of OSA. However, the production of motility appendages, which could protect bacteria from the complement-mediated killing effect, potentially sequestering harmful serum proteins, was never highlighted in genome-wide screens. In contrast, other screens identified the maintenance of lipid asymmetry (*mla*) as a central pathway in serum resistance (73). Mutants in the *mla* locus were also previously identified in *P. aeruginosa* PAO1 strains to be more sensitive to serum killing (74). However, in our screen, the survival of mutants in the MLA pathway was found to be negatively impacted by insertions in *mlaE* and *mlaF* only in the strain YIK (Tables S4 and S5, *mlaE* - Log_2_(FC) = −1.43, *mlaF* - Log_2_(FC) = −1.21).

In this work, we also confirmed that persistence in plasma is a widespread mechanism of *P. aeruginosa* evasion, as resistant strains may become persistent by mutations in their genome. These mutations affecting bacterial surface (e.g., *ssg* mutant) or global metabolism (e.g., *crc* mutant) led to the persistence of a bacterial subpopulation of otherwise plasma-resistant strains. It is worth noting that the complement system is the main immune component responsible for the elimination of *P. aeruginosa* in the blood (9); however, it works in concert with the cellular arm of innate immunity to eliminate invaders. Therefore, genome-wide approaches involving immune cells should be considered for further studies. Given that blood from different donors cannot be pooled, this would require a high number of samples to average the immune variability between individuals. Alternatively, purified innate immune cells together with plasma could be used to screen transposon mutant libraries.

Overall, this work identifies many strain-specific bacterial factors as important in plasma resistance, which constitutes a valuable source of information for future work. This study not only gives insight into a more global comprehension of *P. aeruginosa*/complement system interplay but also raises some concerns about the targeted antimicrobial or anti-virulence strategy, calling for more holistic studies.

## MATERIALS AND METHODS

### Bacterial strains and genetic manipulations

Bacterial cultures were performed in Lysogeny Broth (LB) at 37°C with shaking. LB plates containing irgasan (25 µg/mL) were used to select for *P. aeruginosa*. Antibiotic concentrations used were: 75 µg/mL gentamicin for *P. aeruginosa* and 50 µg/mL gentamicin and 100 µg/mL ampicillin for *E. coli*. All bacterial strains, plasmids, and primers are listed in Tables S9 and S10 Deletion mutants were engineered by amplifying approximately 500–700 bp upstream and downstream flanking regions of the gene of interest, according to (75), from genomic DNA by PCR using appropriate primer pairs (sF1/sR1, sF2/sR2). Subsequently, overlapping fragments were cloned into *Sma*I-digested pEXG2 using Sequence- and Ligation-Independent Cloning (SLIC, [76]). Using triparental mating, allelic exchange vectors were introduced into *P. aeruginosa*, with pRK600 as a helper plasmid. Selection of merodiploid clones resulting from homologous recombination was performed on LB agar plates containing irgasan and gentamicin. Merodiploids colonies were streaked onto NaCl-free LB plates with 10% sucrose (wt/vol) selected for plasmid loss. The resulting clones were screened for gene deletion and antibiotic sensitivity by PCR with appropriate primer pairs.

### Generation of transposon libraries

Libraries were generated as previously described in (21). Briefly, *P. aeruginosa* strains (CHA, YIK, and PA14) were grown overnight from an isolated colony at 37°C with shaking and mixed with *E. coli* SM10 carrying pBTK24 ([33], grown overnight with ampicillin). Biparental conjugation was performed, mixing equal volumes of *P. aeruginosa* and *E. coli* strains. Bacteria were pelleted by centrifugation and resuspended in fresh LB, resulting in a 5-fold concentrated bacterial suspension. Approximately 15 40-µL puddles were spotted onto dry LB plates and incubated at 37°C for 4 h. Bacteria were scraped off the plates and resuspended into LB. Bacterial suspension was then spread onto irgasan- and gentamicin-containing plates and grown at 37°C. Transposon mutant colonies were collected by plate flushing with LB and scraped off using a loop. Glycerol was added to a 20% final concentration. The resulting transposon libraries were aliquoted and stored at −80°C until use.

### Plasma pool preparation

Heparinized human blood from healthy donors was provided by the French National Blood Institute (EFS, Grenoble, France). Fresh blood samples were centrifuged for 10 min at 1,000 × *g* at room temperature. Plasma-containing supernatants of 10 donors were pooled and filtered through a 0.45 µm membrane. Pooled plasma was then aliquoted and stored at −80°C until needed. Prior to use, pooled plasma was thawed at 4°C and centrifuged for 10 min at 10,000 rpm on a microcentrifuge, and the supernatant was filtered through a 0.22-µm membrane. To prepare heat-inactivated plasma (HIP), pooled plasma was thawed at 4°C and inactivated by a 30 min incubation at 56°C followed by a 10 min centrifugation step at 10,000 rpm and filtration through a 0.22-µm membrane.

### Screen in plasma

Screen in plasma was performed as described in (21). Briefly, transposon mutant libraries were thawed on ice and diluted in 30 mL of LB. Bacteria were grown for 16 h at 30°C. Bacterial culture was diluted to OD_600nm_~0.1 and grown at 37°C under agitation. When cultures reached an OD_600nm_~1, bacteria were harvested and resuspended in PBS supplemented with calcium and magnesium ions (PBS +/+, Thermofisher Scientific). Plasma and HIP were inoculated with a final bacterial concentration of 2.25 × 10^7^ CFU/mL (90% final plasma concentration).The initial count was determined by CFU counting, and samples were incubated for 3 h at 37°C on a rotating wheel. Bacterial survival at the end of the incubation was assayed by CFU counting. PA14 was spread onto LB plates to isolate transposon mutants with increased survival in plasma. After the challenge, bacteria were recovered in LB and allowed to grow overnight. Clonal populations of the corresponding wild-type strains were challenged in parallel for survival comparison. About 10^9^ bacteria were harvested for genomic DNA extraction and library constructions.The experimental procedure was performed in biological duplicates.

### Library construction, sequencing, and data analysis

#### Genomic DNA extraction

Bacterial pellets were harvested from the screen and lysed by SDS-NaCl lysis (2% SDS, 0.1M EDTA pH8, 0.6M sodium perchlorate, 0.15M NaCl), followed by two successive steps of phenol-chloroform extractions. Genomic DNA (gDNA) was precipitated in cold ethanol absolute and diluted in the TE buffer (10 mM Tris-HCl pH8, 1 mM EDTA). gDNA was mechanically sheared at 4°C using a QSonica sonicator (Q700, 40% amplitude) for 20 min with 15-s pulses. gDNA fragments, with a size of 150×400 bp, were then concentrated in monarch columns (PCR & DNA cleanup kit, NEB) and controlled on 2% agarose gel.

#### Library construction and sequencing

Fragmented gDNA was end-repaired using an End-repair module (NEB#E6050) followed by a dA-tailing with Klenow (NEB#6053). Annealing of short and long adaptors (Table S10) was performed in a thermocycler with a program allowing a decrease of 1°C/cycle, from 95°C to 20°C. Annealed gDNA fragments were then ligated at 16°C with T4 DNA ligase (NEB#M0202). Fragments of 200×400 bp were selected on a 2% agarose gel and purified from the gel (Monarch DNA Gel Extraction Kit, NEB#T1020L). Purified DNA-adaptor fragments were amplified using the Phusion polymerase (NEB#M0530) and a specific pair of primers (PCR1 Tn-spe direct and PCR1 Adaptor comp.). A second amplification step was performed using primers carrying the P5 and indexed-P7 Illumina sequences (NEB#E7335S). At each step, double-stranded DNA (dsDNA) concentration was assessed using the Qubit dsDNA HS assay kit (Q33230). Finally, the library’s quality was assessed on the Agilent Bioanalyzer 2100 (HS DNA Chips Ref #5067-4626). and sequencing was performed on an Illumina Nextseq High (I2BC, Saclay Paris).

#### Data analysis

Following quality control of the data, sequencing reads were trimmed and aligned to the corresponding genome using Bowtie (77). Read count per feature was calculated by Htseq-count (78), and differential representation of the insertion mutants between the heat-inactivated control and the plasma sample was performed using DESeq2 (79). Concerning the analysis of Tn insertion in the intergenic regions, annotation files were created for each strain attributing the intergenic region to the downstream gene, independent of its orientation. Alignment files produced by Bowtie were then used with these new annotation files and analyzed by Htseq-count and DESeq2. All analyses were performed on the galaxy platform (35). In order to compare data from each strain, a BLAST reciprocal best (80, 81) hit analysis was performed, blasting all the proteins from each strain against PAO1 proteins with a 90% coverage and 80% identity. Reference genomes were downloaded from NCBI; EC2A-CHA: GCF_003698505.1, YIK: JAPTST000000000, UCBPP-PA14: GCF_000014625.1.

### Gene equivalent between strains

Gene equivalents among the strains of interest were determined using Blast Reciprocal Best Hits on Galaxy version 0.3.0 (usegalaxy.eu). Genes were considered equivalent when coverage was higher than 90% and identity higher than 80%.

### Plasma-killing assay

Plasma-killing assays were performed as described in (9). Bacterial cultures were inoculated with an OD_600nm_ of 0.1 and grown at 37°C with shaking until they reached an OD_600nm_ of 1. Bacteria were harvested and resuspended in PBS +/+, and plasma was inoculated with a final bacterial concentration of 2.25 × 10^7^ CFU/mL (90% final plasma concentration). Initial and final bacterial loads were assessed by CFU counting at indicated times.

### LPS extraction and visualization

LPS was extracted by the hot phenol-water method as described previously (82, 83) with some modifications. Briefly, 30 mL of bacterial culture grown in LB to exponential phase (OD_600nm_ =1) of CHA or YIK wild-type or mutants were pelleted by centrifugation and resuspended in 1 mL of 20 mM Tris pH8, 20 mM MgCl_2_, 50 mM NaCl buffer. Sonication was performed for 20 min at 4°C (10 s on/10 s off, 40% amplitude) using a QSonica Oasis-180. In order to eliminate proteins and nucleic acids, treatment with DNase (Euromedex 1307, 50 µg/mL) and RNase (Sigma R6513, 40 µg/mL) was carried out for 1 h 37°C followed by Proteinase K (Roche-311879001) treatment for 1 h at 65°C and an overnight incubation at 37°C. Then, two successive hot phenol-water extractions (Invitrogen 15594-04) were carried out. Briefly, an equal volume was added, incubated for 10 min at 65°C with vigorous shaking every minute, then centrifuged for 10 min at 20,000 × *g* at 4°C. The aqueous phase was precipitated by 0.5M sodium acetate (pH 5.2) with 10 volumes of cold ethanol and stored at −20°C. After centrifugation 10 min 20,000 × *g* at 4°C, the pellets were washed with water and dialyzed. The final purified LPS product was lyophilized and stored at −20°C. Freeze-dried samples were resuspended in SDS-PAGE buffer and boiled for 5 min. Samples were loaded onto a NuPAGE Bis-Tris 4%–12% gel (Invitrogen). LPS were stained using the ProQ Emerald 300 lipopolysaccharide gel stain kit (Molecular Probe P20495).

### CONGO red plates

Five microliters of culture at OD_600nm_ of 1 were spotted on the BHI agar plate (Brain Heart Infusion Broth - Sigma 53286) containing 5% sucrose and 0.8 g/L Congo red (Sigma C6767). Plates were incubated for 24 h at 37°C and at 4°C before imaging.

### Statistical analysis

Statistical analyses were performed using Sigmaplot and Graphpad Prism 9 software. The tests used are specified in figure legends. Survival data were log_10_-transformed. Figures were performed using GraphPad Prism 9 and Inkscape.

## Data Availability

The Tn-seq data generated in this work is available at GEO under the series accession number GSE287536.

## SUPPLEMENTAL MATERIAL

Supplemental material iai.00055-25-s0001.docx Fig. S1 to S4; Tables S1 and S2, S4, S6 and S7, S9 and S10.

Table S8 iai.00055-25-s0002.xlsx Tn-seq data for the strain PA14.

Table S5 iai.00055-25-s0003.xlsx Tn-seq data for the strain YIK.

Table S3 iai.00055-25-s0004.xlsx Tn-seq data for the strain CHA.

ASM does not own the copyrights to Supplemental Material that may be linked to, or accessed through, an article. The authors have granted ASM a non-exclusive, world-wide license to publish the Supplemental Material files. Please contact the corresponding author directly for reuse.

## References

[1] Kang C-I, Kim S-H, Kim H-B, Park S-W, Choe Y-J, Oh M-D, Kim E-C, Choe K-W. 2003. Pseudomonas aeruginosa bacteremia: risk factors for mortality and influence of delayed receipt of effective antimicrobial therapy on clinical outcome. Clin Infect Dis 37:745–751. doi:10.1086/377200 12955633

[2] Vitkauskienė A, Skrodenienė E, Dambrauskienė A, Macas A, Sakalauskas R. 2010. Pseudomonas aeruginosa bacteremia: resistance to antibiotics, risk factors, and patient mortality. Medicina (Kaunas) 46:490–495. doi:10.3390/medicina46070071 20966623

[3] Vallés J, Alvarez-Lerma F, Palomar M, Blanco A, Escoresca A, Armestar F, Sirvent JM, Balasini C, Zaragoza R, Marín M, Study Group of Infectious Diseases of the Spanish Society of Critical Care Medicine. 2011. Health-care-associated bloodstream infections at admission to the ICU. Chest 139:810–815. doi:10.1378/chest.10-1715 21106655

[4] Golovkine G, Faudry E, Bouillot S, Elsen S, Attrée I, Huber P. 2016. Pseudomonas aeruginosa transmigrates at epithelial cell-cell junctions, exploiting sites of cell division and senescent cell extrusion. PLoS Pathog 12:e1005377. doi:10.1371/journal.ppat.1005377 26727615 PMC4699652

[5] Azghani AO. 1996. Pseudomonas aeruginosa and epithelial permeability: role of virulence factors elastase and exotoxin A. Am J Respir Cell Mol Biol 15:132–140. doi:10.1165/ajrcmb.15.1.8679217 8679217

[6] Sana TG, Baumann C, Merdes A, Soscia C, Rattei T, Hachani A, Jones C, Bennett KL, Filloux A, Superti-Furga G, Voulhoux R, Bleves S. 2015. Internalization of Pseudomonas aeruginosa strain PAO1 into epithelial cells is promoted by interaction of a T6SS effector with the microtubule network. mBio 6:e00712. doi:10.1128/mBio.00712-15 26037124 PMC4453011

[7] Vikström E, Tafazoli F, Magnusson K-E. 2006. Pseudomonas aeruginosa quorum sensing molecule N-(3 oxododecanoyl)-l-homoserine lactone disrupts epithelial barrier integrity of Caco-2 cells. FEBS Lett 580:6921–6928. doi:10.1016/j.febslet.2006.11.057 17157842

[8] Pont S, Janet-Maitre M, Faudry E, Cretin F, Attrée I. 2022. Molecular mechanisms involved in *Pseudomonas aeruginosa* bacteremia, p 325–345. In Filloux A, Ramos JL (ed), Pseudomonas aeruginosa: biology, pathogenesis and control strategies. Springer International Publishing, Cham. 10.1007/978-3-031-08491-1_12 36258078

[9] Pont S, Fraikin N, Caspar Y, Van Melderen L, Attrée I, Cretin F. 2020. Bacterial behavior in human blood reveals complement evaders with some persister-like features. PLoS Pathog 16:e1008893. doi:10.1371/journal.ppat.1008893 33326490 PMC7773416

[10] Menny A, Serna M, Boyd CM, Gardner S, Joseph AP, Morgan BP, Topf M, Brooks NJ, Bubeck D. 2018. CryoEM reveals how the complement membrane attack complex ruptures lipid bilayers. Nat Commun 9:5316. doi:10.1038/s41467-018-07653-5 30552328 PMC6294249

[11] Doorduijn DJ, Rooijakkers SHM, Heesterbeek DAC. 2019. How the membrane attack complex damages the bacterial cell envelope and kills Gram-negative bacteria. Bioessays 41:e1900074. doi:10.1002/bies.201900074 31452228

[12] Leduc D, Beaufort N, de Bentzmann S, Rousselle J-C, Namane A, Chignard M, Pidard D. 2007. The Pseudomonas aeruginosa LasB metalloproteinase regulates the human urokinase-type plasminogen activator receptor through domain-specific endoproteolysis. Infect Immun 75:3848–3858. doi:10.1128/IAI.00015-07 17517866 PMC1951998

[13] Laarman AJ, Bardoel BW, Ruyken M, Fernie J, Milder FJ, van Strijp JAG, Rooijakkers SHM. 2012. Pseudomonas aeruginosa alkaline protease blocks complement activation via the classical and lectin pathways. J Immunol 188:386–393. doi:10.4049/jimmunol.1102162 22131330

[14] Nagy ZA, Szakács D, Boros E, Héja D, Vígh E, Sándor N, Józsi M, Oroszlán G, Dobó J, Gál P, Pál G. 2019. Ecotin, a microbial inhibitor of serine proteases, blocks multiple complement dependent and independent microbicidal activities of human serum. PLoS Pathog 15:e1008232. doi:10.1371/journal.ppat.1008232 31860690 PMC6944378

[15] Hallström T, Uhde M, Singh B, Skerka C, Riesbeck K, Zipfel PF. 2015. Pseudomonas aeruginosa uses dihydrolipoamide dehydrogenase (Lpd) to bind to the human terminal pathway regulators vitronectin and clusterin to inhibit terminal pathway complement attack. PLoS One 10:e0137630. doi:10.1371/journal.pone.0137630 26368530 PMC4569481

[16] Kunert A, Losse J, Gruszin C, Hühn M, Kaendler K, Mikkat S, Volke D, Hoffmann R, Jokiranta TS, Seeberger H, Moellmann U, Hellwage J, Zipfel PF. 2007. Immune evasion of the human pathogen Pseudomonas aeruginosa: elongation factor Tuf is a factor H and plasminogen binding protein. J Immunol 179:2979–2988. doi:10.4049/jimmunol.179.5.2979 17709513

[17] Pier GB, Coleman F, Grout M, Franklin M, Ohman DE. 2001. Role of alginate O acetylation in resistance of mucoid Pseudomonas aeruginosa to opsonic phagocytosis. Infect Immun 69:1895–1901. doi:10.1128/IAI.69.3.1895-1901.2001 11179370 PMC98099

[18] Pedersen SS, Kharazmi A, Espersen F, Høiby N. 1990. Pseudomonas aeruginosa alginate in cystic fibrosis sputum and the inflammatory response. Infect Immun 58:3363–3368. doi:10.1128/iai.58.10.3363-3368.1990 2401567 PMC313661

[19] Balaban NQ, Helaine S, Lewis K, Ackermann M, Aldridge B, Andersson DI, Brynildsen MP, Bumann D, Camilli A, Collins JJ, Dehio C, Fortune S, Ghigo J-M, Hardt W-D, Harms A, Heinemann M, Hung DT, Jenal U, Levin BR, Michiels J, Storz G, Tan M-W, Tenson T, Van Melderen L, Zinkernagel A. 2019. Definitions and guidelines for research on antibiotic persistence. Nat Rev Microbiol 17:441–448. doi:10.1038/s41579-019-0196-3 30980069 PMC7136161

[20] Rudra B, Duncan L, Shah AJ, Shah HN, Gupta RS. 2022. Phylogenomic and comparative genomic studies robustly demarcate two distinct clades of Pseudomonas aeruginosa strains: proposal to transfer the strains from an outlier clade to a novel species Pseudomonas paraeruginosa sp. nov. Int J Syst Evol Microbiol 72:005542. doi:10.1099/ijsem.0.005542 36355412

[21] Janet-Maitre M, Pont S, Masson FM, Sleiman S, Trouillon J, Robert-Genthon M, Gallet B, Dumestre-Perard C, Elsen S, Moriscot C, Bardoel BW, Rooijakkers SHM, Cretin F, Attrée I. 2023. Genome-wide screen in human plasma identifies multifaceted complement evasion of Pseudomonas aeruginosa. PLoS Pathog 19:e1011023. doi:10.1371/journal.ppat.1011023 36696456 PMC9901815

[22] Freschi L, Vincent AT, Jeukens J, Emond-Rheault J-G, Kukavica-Ibrulj I, Dupont M-J, Charette SJ, Boyle B, Levesque RC. 2019. The Pseudomonas aeruginosa pan-genome provides new insights on its population structure, horizontal gene transfer, and pathogenicity. Genome Biol Evol 11:109–120. doi:10.1093/gbe/evy259 30496396 PMC6328365

[23] Ozer EA, Nnah E, Didelot X, Whitaker RJ, Hauser AR. 2019. The population structure of Pseudomonas aeruginosa is characterized by genetic isolation of exoU+ and exoS+ lineages. Genome Biol Evol 11:1780–1796. doi:10.1093/gbe/evz119 31173069 PMC6690169

[24] Freschi L, Bertelli C, Jeukens J, Moore MP, Kukavica-Ibrulj I, Emond-Rheault J-G, Hamel J, Fothergill JL, Tucker NP, McClean S, Klockgether J, de Soyza A, Brinkman FSL, Levesque RC, Winstanley C. 2018. Genomic characterisation of an international Pseudomonas aeruginosa reference panel indicates that the two major groups draw upon distinct mobile gene pools. FEMS Microbiol Lett 365. doi:10.1093/femsle/fny120 29897457

[25] Hilker R, Munder A, Klockgether J, Losada PM, Chouvarine P, Cramer N, Davenport CF, Dethlefsen S, Fischer S, Peng H, Schönfelder T, Türk O, Wiehlmann L, Wölbeling F, Gulbins E, Goesmann A, Tümmler B. 2015. Interclonal gradient of virulence in the Pseudomonas aeruginosa pangenome from disease and environment. Environ Microbiol 17:29–46. doi:10.1111/1462-2920.12606 25156090

[26] Toussaint B, Delic-Attree I, Vignais PM. 1993. Pseudomonas aeruginosa contains an IHF-like protein that binds to the algD promoter. Biochem Biophys Res Commun 196:416–421. doi:10.1006/bbrc.1993.2265 8216322

[27] Bezuidt OK, Klockgether J, Elsen S, Attree I, Davenport CF, Tümmler B. 2013. Intraclonal genome diversity of Pseudomonas aeruginosa clones CHA and TB. BMC Genomics 14:416. doi:10.1186/1471-2164-14-416 23799896 PMC3697988

[28] Elabbadi A, Pont S, Verdet C, Plésiat P, Cretin F, Voiriot G, Fartoukh M, Djibré M. 2020. An unusual community-acquired invasive and multi systemic infection due to ExoU-harboring Pseudomonas aeruginosa strain: clinical disease and microbiological characteristics. J Microbiol Immunol Infect 53:647–651. doi:10.1016/j.jmii.2019.06.008 31345686

[29] Rahme LG, Stevens EJ, Wolfort SF, Shao J, Tompkins RG, Ausubel FM. 1995. Common virulence factors for bacterial pathogenicity in plants and animals. Science 268:1899–1902. doi:10.1126/science.7604262 7604262

[30] Trouillon J, Sentausa E, Ragno M, Robert-Genthon M, Lory S, Attrée I, Elsen S. 2020. Species-specific recruitment of transcription factors dictates toxin expression. Nucleic Acids Res 48:2388–2400. doi:10.1093/nar/gkz1232 31925438 PMC7049718

[31] Kos VN, Déraspe M, McLaughlin RE, Whiteaker JD, Roy PH, Alm RA, Corbeil J, Gardner H. 2015. The resistome of Pseudomonas aeruginosa in relationship to phenotypic susceptibility. Antimicrob Agents Chemother 59:427–436. doi:10.1128/AAC.03954-14 25367914 PMC4291382

[32] Hao Y, Murphy K, Lo RY, Khursigara CM, Lam JS. 2015. Single-nucleotide polymorphisms found in the migA and wbpX glycosyltransferase genes account for the intrinsic lipopolysaccharide defects exhibited by Pseudomonas aeruginosa PA14. J Bacteriol 197:2780–2791. doi:10.1128/JB.00337-15 26078447 PMC4524037

[33] Kulasekara HD, Ventre I, Kulasekara BR, Lazdunski A, Filloux A, Lory S. 2005. A novel two-component system controls the expression of Pseudomonas aeruginosa fimbrial cup genes. Mol Microbiol 55:368–380. doi:10.1111/j.1365-2958.2004.04402.x 15659157

[34] Poulsen BE, Yang R, Clatworthy AE, White T, Osmulski SJ, Li L, Penaranda C, Lander ES, Shoresh N, Hung DT. 2019. Defining the core essential genome of Pseudomonas aeruginosa. Proc Natl Acad Sci USA 116:10072–10080. doi:10.1073/pnas.1900570116 31036669 PMC6525520

[35] Afgan E, Baker D, Batut B, van den Beek M, Bouvier D, Cech M, Chilton J, Clements D, Coraor N, Grüning BA, Guerler A, Hillman-Jackson J, Hiltemann S, Jalili V, Rasche H, Soranzo N, Goecks J, Taylor J, Nekrutenko A, Blankenberg D. 2018. The Galaxy platform for accessible, reproducible and collaborative biomedical analyses: 2018 update. Nucleic Acids Res 46:W537–W544. doi:10.1093/nar/gky379 29790989 PMC6030816

[36] Zhou C, Dadashi M, Wu M. 2020. Expanding roles and regulatory networks of LadS/RetS in *Pseudomonas aeruginosa*. In Trends in quorum sensing and quorum quenching. CRC Press.

[37] Gebhardt MJ, Kambara TK, Ramsey KM, Dove SL. 2020. Widespread targeting of nascent transcripts by RsmA in Pseudomonas aeruginosa. Proc Natl Acad Sci USA 117:10520–10529. doi:10.1073/pnas.1917587117 32332166 PMC7229658

[38] Mishra M, Ressler A, Schlesinger LS, Wozniak DJ. 2015. Identification of OprF as a complement component C3 binding acceptor molecule on the surface of Pseudomonas aeruginosa. Infect Immun 83:3006–3014. doi:10.1128/IAI.00081-15 25964476 PMC4496607

[39] Short FL, Di Sario G, Reichmann NT, Kleanthous C, Parkhill J, Taylor PW. 2020. Genomic profiling reveals distinct routes to complement resistance in Klebsiella pneumoniae. Infect Immun 88:e00043-20. doi:10.1128/IAI.00043-20 32513855 PMC7375759

[40] Mishra M, Byrd MS, Sergeant S, Azad AK, Parsek MR, McPhail L, Schlesinger LS, Wozniak DJ. 2012. Pseudomonas aeruginosa Psl polysaccharide reduces neutrophil phagocytosis and the oxidative response by limiting complement-mediated opsonization. Cell Microbiol 14:95–106. doi:10.1111/j.1462-5822.2011.01704.x 21951860 PMC4466118

[41] Wu H, Wang D, Tang M, Ma LZ. 2019. The advance of assembly of exopolysaccharide Psl biosynthesis machinery in Pseudomonas aeruginosa. Microbiologyopen 8:e857. doi:10.1002/mbo3.857 31070012 PMC6813494

[42] Franklin MJ, Nivens DE, Weadge JT, Howell PL. 2011. Biosynthesis of the Pseudomonas aeruginosa extracellular polysaccharides, alginate, Pel, and Psl. Front Microbiol 2:167. doi:10.3389/fmicb.2011.00167 21991261 PMC3159412

[43] Jones CJ, Wozniak DJ. 2017. Psl produced by mucoid Pseudomonas aeruginosa contributes to the establishment of biofilms and immune evasion. mBio 8:e00864-17. doi:10.1128/mBio.00864-17 28634241 PMC5478896

[44] Brencic A, McFarland KA, McManus HR, Castang S, Mogno I, Dove SL, Lory S. 2009. The GacS/GacA signal transduction system of Pseudomonas aeruginosa acts exclusively through its control over the transcription of the RsmY and RsmZ regulatory small RNAs. Mol Microbiol 73:434–445. doi:10.1111/j.1365-2958.2009.06782.x 19602144 PMC2761719

[45] Mikkelsen H, McMullan R, Filloux A. 2011. The Pseudomonas aeruginosa reference strain PA14 displays increased virulence due to a mutation in ladS. PLoS One 6:e29113. doi:10.1371/journal.pone.0029113 22216178 PMC3245244

[46] Colvin KM, Gordon VD, Murakami K, Borlee BR, Wozniak DJ, Wong GCL, Parsek MR. 2011. The Pel polysaccharide can serve a structural and protective role in the biofilm matrix of Pseudomonas aeruginosa. PLoS Pathog 7:e1001264. doi:10.1371/journal.ppat.1001264 21298031 PMC3029257

[47] Wozniak DJ, Wyckoff TJO, Starkey M, Keyser R, Azadi P, O’Toole GA, Parsek MR. 2003. Alginate is not a significant component of the extracellular polysaccharide matrix of PA14 and PAO1 Pseudomonas aeruginosa biofilms. Proc Natl Acad Sci USA 100:7907–7912. doi:10.1073/pnas.1231792100 12810959 PMC164686

[48] Hershberger CD, Ye RW, Parsek MR, Xie ZD, Chakrabarty AM. 1995. The algT (algU) gene of Pseudomonas aeruginosa, a key regulator involved in alginate biosynthesis, encodes an alternative sigma factor (sigma E). Proc Natl Acad Sci USA 92:7941–7945. doi:10.1073/pnas.92.17.7941 7644517 PMC41262

[49] Jones AK, Fulcher NB, Balzer GJ, Urbanowski ML, Pritchett CL, Schurr MJ, Yahr TL, Wolfgang MC. 2010. Activation of the Pseudomonas aeruginosa AlgU regulon through mucA mutation inhibits cyclic AMP/Vfr signaling. J Bacteriol 192:5709–5717. doi:10.1128/JB.00526-10 20817772 PMC2953679

[50] Lizewski SE, Lundberg DS, Schurr MJ. 2002. The transcriptional regulator AlgR is essential for Pseudomonas aeruginosa pathogenesis. Infect Immun 70:6083–6093. doi:10.1128/IAI.70.11.6083-6093.2002 12379685 PMC130412

[51] Lizewski SE, Schurr JR, Jackson DW, Frisk A, Carterson AJ, Schurr MJ. 2004. Identification of AlgR-regulated genes in Pseudomonas aeruginosa by use of microarray analysis. J Bacteriol 186:5672–5684. doi:10.1128/JB.186.17.5672-5684.2004 15317771 PMC516850

[52] Bucior I, Pielage JF, Engel JN. 2012. Pseudomonas aeruginosa pili and flagella mediate distinct binding and signaling events at the apical and basolateral surface of airway epithelium. PLoS Pathog 8:e1002616. doi:10.1371/journal.ppat.1002616 22496644 PMC3320588

[53] Homma M, Kutsukake K, Hasebe M, Iino T, Macnab RM. 1990. FlgB, FlgC, FlgF and FlgG: a family of structurally related proteins in the flagellar basal body of Salmonella typhimurium. J Mol Biol 211:465–477. doi:10.1016/0022-2836(90)90365-S 2129540

[54] Koo J, Tang T, Harvey H, Tammam S, Sampaleanu L, Burrows LL, Howell PL. 2013. Functional mapping of PilF and PilQ in the Pseudomonas aeruginosa type IV pilus system. Biochemistry 52:2914–2923. doi:10.1021/bi3015345 23547883

[55] Chang Y-S, Klockgether J, Tümmler B. 2007. An intragenic deletion in pilQ leads to nonpiliation of a Pseudomonas aeruginosa strain isolated from cystic fibrosis lung. FEMS Microbiol Lett 270:201–206. doi:10.1111/j.1574-6968.2007.00664.x 17302936

[56] Chen Y-H, Li S-H, Yang Y-C, Hsu S-H, Nizet V, Chang Y-C. 2020. T4 pili promote colonization and immune evasion phenotypes of nonencapsulated M4 Streptococcus pyogenes. mBio 11:e01580. doi:10.1128/mBio.01580-20 32694142 PMC7374061

[57] Duong-Nu T-M, Jeong K, Hong SH, Puth S, Kim SY, Tan W, Lee KH, Lee SE, Rhee JH. 2019. A stealth adhesion factor contributes to Vibrio vulnificus pathogenicity: Flp pili play roles in host invasion, survival in the blood stream and resistance to complement activation. PLoS Pathog 15:e1007767. doi:10.1371/journal.ppat.1007767 31437245 PMC6748444

[58] Yin L, Shen X, Zhang D, Zhao R, Dai Y, Hu X, Zhou X, Hou H, Pan X, Qi K. 2021. Flagellar rotor protein FliG is involved in the virulence of avian pathogenic Escherichia coli. Microb Pathog 160:105198. doi:10.1016/j.micpath.2021.105198 34537273

[59] Kintz E, Scarff JM, DiGiandomenico A, Goldberg JB. 2008. Lipopolysaccharide O-antigen chain length regulation in Pseudomonas aeruginosa serogroup O11 strain PA103. J Bacteriol 190:2709–2716. doi:10.1128/JB.01646-07 18065548 PMC2293223

[60] Lam JS, Taylor VL, Islam ST, Hao Y, Kocíncová D. 2011. Genetic and functional diversity of Pseudomonas aeruginosa lipopolysaccharide. Front Microbiol 2:118. doi:10.3389/fmicb.2011.00118 21687428 PMC3108286

[61] Rocchetta HL, Burrows LL, Lam JS. 1999. Genetics of O-antigen biosynthesis in Pseudomonas aeruginosa. Microbiol Mol Biol Rev 63:523–553. doi:10.1128/MMBR.63.3.523-553.1999 10477307 PMC103745

[62] Cross AR, Goldberg JB. 2019. Remodeling of O antigen in mucoid Pseudomonas aeruginosa via transcriptional repression of wzz2. mBio 10:e02914-18. doi:10.1128/mBio.02914-18 30782665 PMC6381286

[63] Kintz EN, Goldberg JB. 2011. Site-directed mutagenesis reveals key residue for O antigen chain length regulation and protein stability in Pseudomonas aeruginosa Wzz2. J Biol Chem 286:44277–44284. doi:10.1074/jbc.M111.273979 22069314 PMC3243511

[64] Veeranagouda Y, Lee K, Cho AR, Cho K, Anderson EM, Lam JS. 2011. Ssg, a putative glycosyltransferase, functions in lipo- and exopolysaccharide biosynthesis and cell surface-related properties in Pseudomonas alkylphenolia. FEMS Microbiol Lett 315:38–45. doi:10.1111/j.1574-6968.2010.02172.x 21166709

[65] Azimi S, Thomas J, Cleland SE, Curtis JE, Goldberg JB, Diggle SP. 2021. O-specific antigen-dependent surface hydrophobicity mediates aggregate assembly type in Pseudomonas aeruginosa. mBio 12:e0086021. doi:10.1128/mBio.00860-21 34372703 PMC8406328

[66] Li Y, Xia H, Bai F, Song X, Zhuang L, Xu H, Zhang X, Zhang X, Qiao M. 2020. PA5001 gene involves in swimming motility and biofilm formation in Pseudomonas aeruginosa. Microb Pathog 144:103982. doi:10.1016/j.micpath.2020.103982 32105802

[67] Fernández L, Álvarez-Ortega C, Wiegand I, Olivares J, Kocíncová D, Lam JS, Martínez JL, Hancock REW. 2013. Characterization of the polymyxin B resistome of Pseudomonas aeruginosa. Antimicrob Agents Chemother 57:110–119. doi:10.1128/AAC.01583-12 23070157 PMC3535977

[68] Linares JF, Moreno R, Fajardo A, Martínez-Solano L, Escalante R, Rojo F, Martínez JL. 2010. The global regulator Crc modulates metabolism, susceptibility to antibiotics and virulence in Pseudomonas aeruginosa. Environ Microbiol 12:3196–3212. doi:10.1111/j.1462-2920.2010.02292.x 20626455

[69] Ghafoor A, Hay ID, Rehm BHA. 2011. Role of exopolysaccharides in Pseudomonas aeruginosa biofilm formation and architecture. Appl Environ Microbiol 77:5238–5246. doi:10.1128/AEM.00637-11 21666010 PMC3147449

[70] Patarakul K, Cole MF, Hughes CAN. 1999. Complement resistance in Borrelia burgdorferi strain 297: outer membrane proteins prevent MAC formation at lysis susceptible sites. Microb Pathog 27:25–41. doi:10.1006/mpat.1999.0280 10371707

[71] Doorduijn DJ, Rooijakkers SHM, van Schaik W, Bardoel BW. 2016. Complement resistance mechanisms of Klebsiella pneumoniae. Immunobiology 221:1102–1109. doi:10.1016/j.imbio.2016.06.014 27364766

[72] Heesterbeek DAC, Martin NI, Velthuizen A, Duijst M, Ruyken M, Wubbolts R, Rooijakkers SHM, Bardoel BW. 2019. Complement-dependent outer membrane perturbation sensitizes Gram-negative bacteria to Gram-positive specific antibiotics. Sci Rep 9:3074. doi:10.1038/s41598-019-38577-9 30816122 PMC6395757

[73] Sanchez-Larrayoz AF, Elhosseiny NM, Chevrette MG, Fu Y, Giunta P, Spallanzani RG, Ravi K, Pier GB, Lory S, Maira-Litrán T. 2017. Complexity of complement resistance factors expressed by Acinetobacter baumannii needed for survival in human serum. J Immunol 199:2803–2814. doi:10.4049/jimmunol.1700877 28855313 PMC5636677

[74] Munguia J, LaRock DL, Tsunemoto H, Olson J, Cornax I, Pogliano J, Nizet V. 2017. The Mla pathway is critical for Pseudomonas aeruginosa resistance to outer membrane permeabilization and host innate immune clearance. J Mol Med (Berl) 95:1127–1136. doi:10.1007/s00109-017-1579-4 28844103 PMC5671890

[75] Hmelo LR, Borlee BR, Almblad H, Love ME, Randall TE, Tseng BS, Lin C, Irie Y, Storek KM, Yang JJ, Siehnel RJ, Howell PL, Singh PK, Tolker-Nielsen T, Parsek MR, Schweizer HP, Harrison JJ. 2015. Precision-engineering the Pseudomonas aeruginosa genome with two-step allelic exchange. Nat Protoc 10:1820–1841. doi:10.1038/nprot.2015.115 26492139 PMC4862005

[76] Li MZ, Elledge SJ. 2007. Harnessing homologous recombination in vitro to generate recombinant DNA via SLIC. Nat Methods 4:251–256. doi:10.1038/nmeth1010 17293868

[77] Langmead B, Salzberg SL. 2012. Fast gapped-read alignment with Bowtie 2. Nat Methods 9:357–359. doi:10.1038/nmeth.1923 22388286 PMC3322381

[78] Anders S, Pyl PT, Huber W. 2015. HTSeq—a Python framework to work with high-throughput sequencing data. Bioinformatics 31:166–169. doi:10.1093/bioinformatics/btu638 25260700 PMC4287950

[79] Love MI, Huber W, Anders S. 2014. Moderated estimation of fold change and dispersion for RNA-seq data with DESeq2. Genome Biol 15:550. doi:10.1186/s13059-014-0550-8 25516281 PMC4302049

[80] Cock PJA, Chilton JM, Grüning B, Johnson JE, Soranzo N. 2015. NCBI BLAST+ integrated into Galaxy. Gigascience 4:39. doi:10.1186/s13742-015-0080-7 26336600 PMC4557756

[81] Camacho C, Coulouris G, Avagyan V, Ma N, Papadopoulos J, Bealer K, Madden TL. 2009. BLAST+: architecture and applications. BMC Bioinformatics 10:421. doi:10.1186/1471-2105-10-421 20003500 PMC2803857

[82] Rezania S, Amirmozaffari N, Tabarraei B, Jeddi-Tehrani M, Zarei O, Alizadeh R, Masjedian F, Zarnani AH. 2011. Extraction, purification and characterization of lipopolysaccharide from Escherichia coli and Salmonella typhi. Avicenna J Med Biotechnol 3:3–9. 23407691 PMC3558174

[83] Davis MR, Goldberg JB. 2012. Purification and visualization of lipopolysaccharide from Gram-negative bacteria by hot aqueous-phenol extraction. J Vis Exp 3916:3916. doi:10.3791/3916 PMC3466933 22688346

