# Supplementary material for: Strain-specific variation in the complement resistome of Pseudomonas aeruginosa
Source: Infect Immun. 2025 Aug 11;93(9):e00055-25. doi: 10.1128/iai.00055-25 (PMC12418747; doi:10.1128/iai.00055-25)
Supplement: Supplemental material — Fig. S1 to S4; Tables S1 and S2, S4, S6 and S7, S9 and S10. [file iai.00055-25-s0001.docx]

**Strain-Specific Variation in the Complement Resistome of *Pseudomonas aeruginosa***

Manon Janet-Maitre^1*#^, Mylène Robert-Genthon^1^, François Cretin^1^, Sylvie Elsen^1^ and Ina Attrée^1#^

^1^ University Grenoble Alpes, Institut de Biologie Structurale - IBS, UMR5075, Team Bacterial Pathogenesis and Cellular Responses, 38054 Grenoble, France

* Current address: Department of Molecular Microbiology, Washington University School of Medicine, St. Louis, Missouri, USA

^#^ Corresponding authors: [ina.attree@ibs.fr](mailto:ina.attree@ibs.fr), [manon@wustl.edu](mailto:manon@wustl.edu)

**Supplementary material**


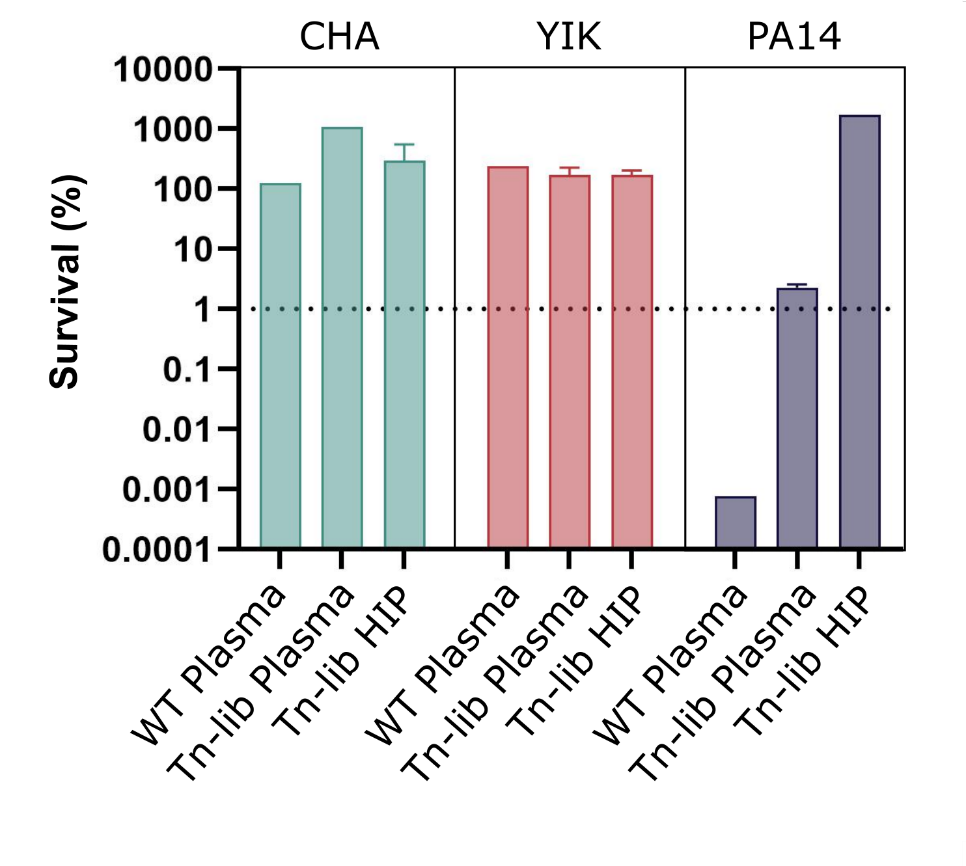


**Figure S1: Plasma sensitivity during the transposon mutant screen.** Survival after 3h-incubation in human pooled plasma or heat-inactivated plasma (HIP) was calculated for each WT and the pooled Tn-library during the screen, using colony forming units (CFU) measurement.

**
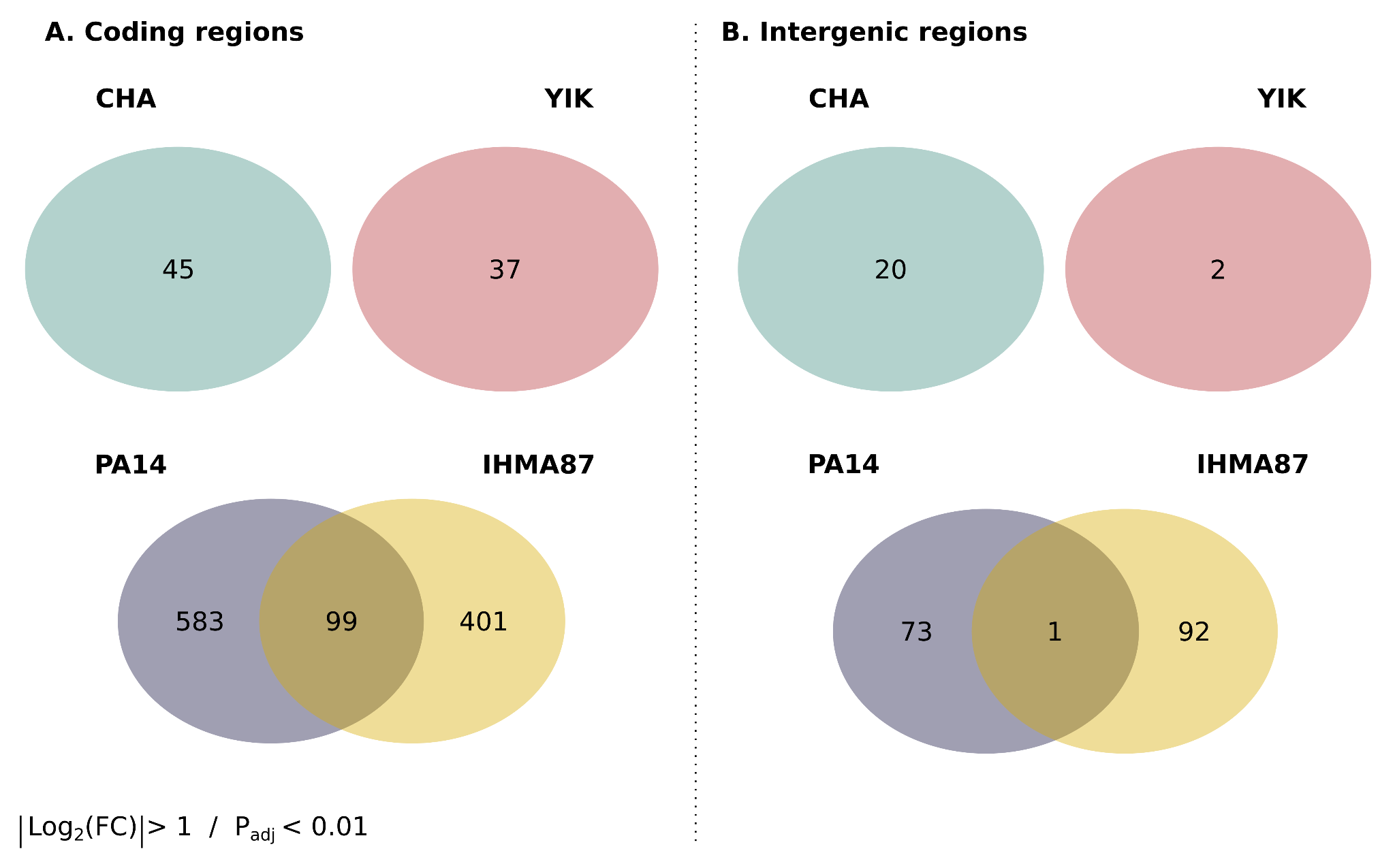
**

**Figure S2. Venn diagram from data in the four strains.** Venn diagram representation of overlapping hits with a |Log_2_(FC)|>1 / P_adj_ <0.01. Gene homology was performed by BLAST reciprocal best hit with PAO1 (Coverage 90%, identity 80 %). Data from IHMA87 was taken from (1).

**
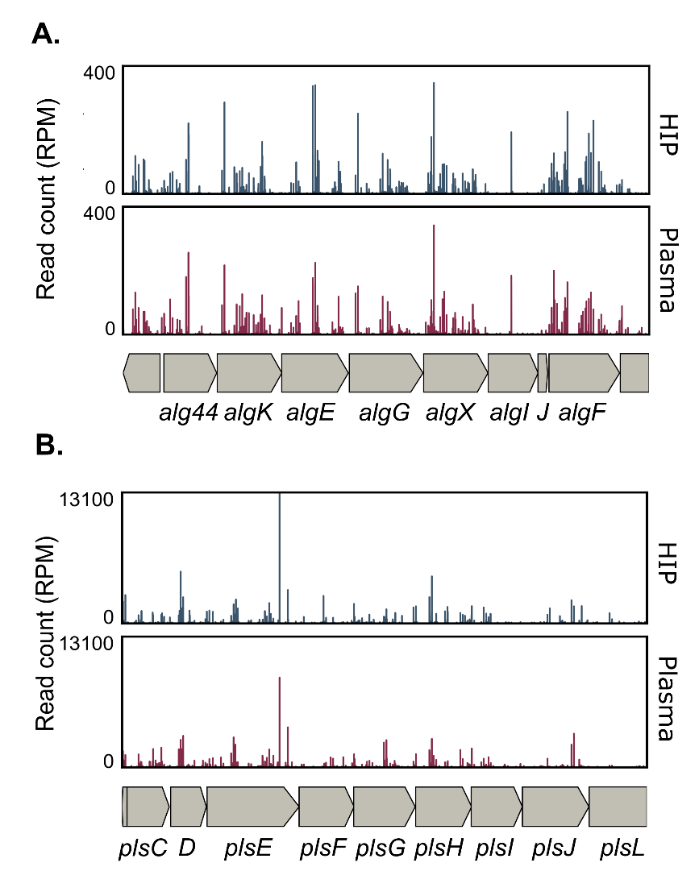
**

**Figure S3. The plasma resistance of CHA transposon insertions mutants in the *alg* and *psl* operons is unchanged.** Tn-seq profiles of *alg* (**A.**) and *psl* (**B.**) operons in CHA, showing number of normalized reads in input (HIP) and output (plasma).

**
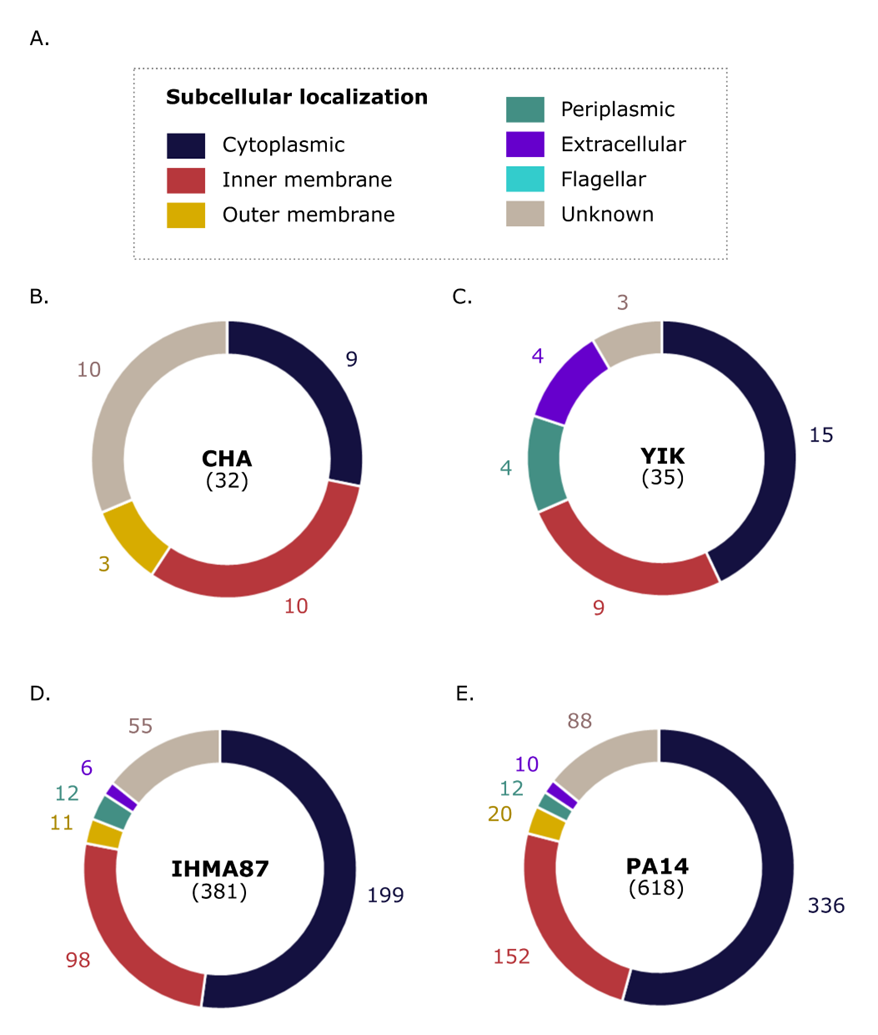

Figure S4. Predicted cellular localization of significant hits from the Tn-seq experiment.** BLAST reciprocal best hit analysis (2) was performed to identify homologs of PAO1 proteins and annotations of PAO1 protein subcellular localization available on *Pseudomonas* Genome database (3) were applied to the significant hits. Hits were considered as significant if Log_2_(FC)>1 and P_adj_<0.01. Possible subcellular localizations are presented in (A), and subcellular localizations of significant hits are depicted for CHA (B), YIK (C), IHMA87 (D), PA14 (E). Data from IHMA87 was taken from (1)

| Strain | PA14 | CHA | YIK |
| --- | --- | --- | --- |
| Total number of genes | 5983 | 6125 | 5821 |
| Genes with no Tn insertion  < 10 sequencing reads | 519 | 1595 | 861 |
| Genes with no Tn insertion  0 sequencing reads | 181 | 985 | 203 |
| Number of genes targeted  At least one Tn insertion | 5464 / 91.3 % | 4530 / 74.0 % | 4960 / 85.2 % |

**Table S1: Most *P. aeruginosa* genes were targeted during Tn-seq.** A gene was considered as targeted by at least one transposon when more than 10 sequencing reads were assigned to this gene in the input (inactivated plasma) sequencing data.

**Table S2. Top hits of insertion mutants in CHA genes decreased in plasma compared to heat inactivated plasma.**

** IT/S/VT=Intracellular trafficking, secretion, and vesicular transport, Rep./Rec./Repair= Replication, recombination and repair, Signal Transduc= Signal transduction mechanisms*

***Predicted function and subcellular localization based on homology with PAO1 genes* (3)

****Intergenic regions (IG) were assigned to the gene upstream independently of its orientation*

| **Rank** | **CHA_ID** | **PA14_ID** | ***Name*** | **Predicted function*, **** | **Log_2_(FC)** | **P_adj_** | **Subcellular localization**** |
| --- | --- | --- | --- | --- | --- | --- | --- |
| 1 | *RS23815* |  | *wzy* |  | -5.75 | 5.70E-110 | NA |
| 2 | *RS13055* |  | *wzz* |  | -3.71 | 5.07E-30 | NA |
| 3 | *RS08345* | PA14_70390 | *crc* | Rep./Rec./Repair | -2.86 | 1.94E-24 | Cytoplasmic |
| 4 | *RS27320* | PA14_57100 | *ampG* | Function unknown | -2.82 | 1.54E-34 | IM |
| 5 | *RS08720* | PA14_69470 | *algR* | Transcription | -2.69 | 3.07E-32 | Cytoplasmic |
| 6 | *RS28395* | PA14_54430 | *algU* | Transcription | -2.67 | 3.07E-32 | Cytoplasmic |
| 7 | *RS29180* |  | *fimU* | IT/S/VT | -2.60 | 4.66E-31 | Unknown |
| 8 | *RS12025* | PA14_25770 | *pilZ* | IT/S/VT | -2.50 | 1.40E-22 | Unknown |
| 9 | *RS24600* | PA14_71920 | *wbpY* | Envelope biogenesis | -2.45 | 3.03E-11 | Cytoplasmic |
| 10 | *RS24955* |  |  |  | -2.43 | 2.11E-26 | NA |
| 11 | *RS29050* | PA14_58770 | *pilD* | Cell motility | -2.38 | 1.40E-22 | IM |
| 12 | *RS09930* | PA14_66640 | *pilO* | Cell motility | -2.31 | 6.53E-21 | IM |
| 13 | *RS09935* | PA14_66630 | *pilP* | IT/S/VT | -2.29 | 2.99E-18 | IM |
| 14 | *RS29190* |  | *pilW* | IT/S/VT | -2.29 | 5.07E-30 | Unknown |
| 15 | *RS29185* |  | *pilV* | IT/S/VT | -2.27 | 3.33E-17 | Unknown |
| 16 | *RS29200* |  | *pilY1* | Cell motility | -2.27 | 8.29E-18 | OM |
| 17 | *RS23700* | PA14_20810 | *recQ* | Transcription | -2.26 | 3.16E-27 | Cytoplasmic |
| 18 | *RS29210* |  | *pilE* | IT/S/VT | -2.24 | 9.29E-16 | Unknown |
| 19 | *RS29040* | PA14_58750 | *pilB* | IT/S/VT | -2.22 | 7.22E-16 | Cytoplasmic |
| 20 | *RS22220* |  |  |  | -2.21 | 2.69E-12 | NA |
| 21 | *RS29195* |  | *pilX* | IT/S/VT | -2.21 | 1.07E-20 | Unknown |
| 22 | *RS29045* |  |  |  | -2.17 | 9.65E-14 | NA |
| 23 | *RS09925* | PA14_66650 | *pilN* | IT/S/VT | -2.17 | 7.90E-19 | IM |
| 24 | *RS09920* | PA14_66660 | *pilM* | Cell motility | -2.15 | 2.06E-12 | IM |
| 25 | *RS13035* |  |  |  | -2.14 | 7.09E-08 | NA |
| 26 | *RS12850* | PA14_23830 | *fimV* | IT/S/VT | -2.14 | 1.07E-13 | Unknown |
| 27 | *RS09940* | PA14_66620 | *pilQ* | IT/S/VT | -2.11 | 2.15E-12 | OM |
| 28 | *RS10370* | PA14_65540 | *fimX* | Signal transduc. | -2.08 | 1.34E-15 | IM |
| 29 | *RS01875* | PA14_14850 | *pilF* | Cell motility | -2.07 | 1.39E-12 | OM |
| 30 | *RS29165* | PA14_60260 | *pilR* | Signal transduc. | -2.04 | 2.61E-14 | Cytoplasmic |
| 31 | *RS22195* | PA14_48940 | *coaB* | Function unknown | -1.92 | 2.20E-15 | IM |
| 32 | *RS22225* |  |  |  | -1.85 | 5.98E-15 | NA |
| 33 | *RS05710* |  |  |  | -1.73 | 7.77E-13 | NA |
| 34 | *RS29035* |  |  |  | -1.67 | 2.99E-09 | NA |
| 35 | *RS22210* |  |  |  | -1.64 | 5.31E-07 | NA |
| 36 | *RS29030* |  |  |  | -1.61 | 3.57E-05 | NA |
| 37 | *RS05730* | PA14_48990 |  | Function unknown | -1.60 | 3.47E-04 | Unknown |
| 38 | *RS05720* | PA14_48970 |  | Function unknown | -1.51 | 1.89E-10 | Unknown |
| 39 | *RS08985* | PA14_68820 |  | IT/S/VT | -1.44 | 5.94E-09 | Cytoplasmic |
| 40 | *RS12020* | PA14_25780 |  | Rep./Rec./Repair | -1.35 | 1.90E-06 | Cytoplasmic |
| **Rank** | **CHA_ID_IG*** | **PA14_ID_IG** | ***Name*** | **Predicted function**** | **Log_2_(FC)** | **P_adj_** |  |
| 1 | RS10185_IG | PA14_66050_IG |  | Lipid transport and metabolism | -3.30 | 3.84E-06 |  |
| 2 | RS05730_IG | PA14_48990_IG |  | Function unknown | -2.83 | 3.75E-05 |  |
| 3 | RS23820_IG | PA14_04340_IG |  | Carbohydrate transport and metabolism | -2.83 | 3.21E-13 |  |
| 4 | RS12025_IG | PA14_25770_IG | *pilZ* | Intracellular trafficking, secretion, and vesicular transport | -2.69 | 1.79E-21 |  |
| 5 | RS05710_IG | PA14_13450_IG |  | Signal transduction mechanisms | -2.67 | 2.35E-05 |  |
| 6 | RS29200_IG |  | *pilY1* | Cell motility | -2.29 | 3.27E-04 |  |
| 7 | RS10985_IG |  |  | Signal transduction mechanisms | -2.28 | 7.96E-16 |  |
| 8 | RS10720_IG | PA14_51990_IG | *wrbA* | General function prediction only | -2.19 | 3.33E-12 |  |
| 9 | RS12855_IG | PA14_23810_IG |  | Amino acid transport and metabolism | -2.17 | 2.84E-20 |  |
| 10 | RS28395_IG | PA14_54430_IG | *algU* | Transcription | -2.14 | 1.10E-11 |  |
| 11 | RS29045_IG | PA14_41270_IG | *parS* | Signal transduction mechanisms | -2.09 | 3.10E-03 |  |
| 12 | RS22195_IG | PA14_48940_IG | *coaB* | Function unknown | -2.04 | 1.37E-08 |  |
| 13 | RS09920_IG | PA14_66660_IG | *pilM* | Cell motility | -1.95 | 2.08E-03 |  |
| 14 | RS17665_IG | PA14_41980_IG |  | Function unknown | -1.92 | 1.97E-04 |  |
| 15 | RS29180_IG |  | *fimU* | Intracellular trafficking, secretion, and vesicular transport | -1.92 | 9.13E-06 |  |
| 16 | RS05725_IG | PA14_48980_IG |  | Function unknown | -1.90 | 3.46E-02 |  |
| 17 | RS07970_IG | PA14_52610_IG |  | Amino acid transport and metabolism | -1.79 | 5.35E-03 |  |
| 18 | RS29035 | PA14_41160_IG |  | General function prediction only | -1.78 | 1.30E-08 |  |
| 19 | RS28105_IG | PA14_55170_IG | *cat* | General function prediction only | -1.56 | 6.82E-04 |  |
| 20 | RS05705_IG | PA14_13520_IG |  | Envelope biogenesis | -1.46 | 5.88E-06 |  |
| 21 | RS07150_IG | PA14_45350_IG | *ccmC* | Posttranslational modification, protein turnover, chaperones | -1.20 | 1.76E-03 |  |
| 22 | RS01685_IG | PA14_17780_IG |  | General function prediction only | -1.07 | 1.08E-02 |  |
| 23 | RS28425_IG | PA14_54350_IG | *lepB* | Intracellular trafficking, secretion, and vesicular transport | 2.02 | 3.63E-02 |  |
| 24 | RS03950_IG | PA14_09680_IG | *bfiS* | Signal transduction mechanisms | 2.26 | 2.75E-02 |  |

**Table S3. Full Tn-seq data in the strain CHA.**

**Table S4. Top hits of insertion mutants in YIK genes and intergenic regions decreased in plasma compared to heat inactivated plasma.**

**Predicted function and subcellular localization based on homology with PAO1 genes* (3)*, IT/S/VT=Intracellular trafficking, secretion, and vesicular transport*

***Intergenic regions (IG) were assigned to the gene upstream independently of its orientation*

*.*

| **Rank** | **YIK_ID** | **PA14_ID** | ***Name*** | **Predicted function*** | **Log_2_(FC)** | **P_adj_** | **Subcellular localization*** |
| --- | --- | --- | --- | --- | --- | --- | --- |
| 1 | *02677* | PA14_35690 | *pslE* | Envelope biogenesis | -5.33 | 3.68E-67 | Cytoplasmic |
| 2 | *00322* | PA14_66120 | *ssg* | Function unknown | -4.31 | 1.30E-56 | IM |
| 3 | *05502* |  | *lppC* |  | -3.87 | 1.04E-29 | Periplasmic |
| 4 | *02674* | PA14_35650 | *pslH* | Envelope biogenesis | -2.95 | 1.38E-16 | Periplasmic |
| 5 | *02370* | PA14_42970 | *sfa2* | Transcription | -2.10 | 1.96E-09 | Extracellular |
| 6 | *03637* | PA14_52260 | *gacS* | Signal transd. | -1.89 | 2.90E-07 | Periplasmic |
| 7 | *02678* |  | *pslD* | Envelope biogenesis | -1.86 | 8.34E-06 | Extracellular |
| 8 | *00606* | PA14_62620 | *pgi* | Carbohydrate metabo. | -1.85 | 4.69E-05 | Extracellular |
| 9 | *01606* | PA14_50240 |  | Function unknown | -1.84 | 9.65E-06 | Unknown |
| 10 | *03840* | PA14_30650 | *gacA* | Transcription | -1.84 | 1.33E-06 | IM |
| 11 | *00988* | PA14_23530 |  | Defense mechanisms | -1.83 | 1.33E-06 | Cytoplasmic |
| 12 | *04706* | PA14_70930 | *yciA* | Lipid | -1.72 | 3.27E-09 | Periplasmic |
| 13 | *02585* | PA14_40380 |  | Transcription | -1.70 | 6.43E-04 | Flagellar |
| 14 | *01263* | PA14_45720 | *flhB* | Cell motility | -1.64 | 5.27E-07 | IM |
| 15 | *01621* | PA14_50440 | *flgF* | Cell motility | -1.64 | 2.35E-06 | IM |
| 16 | *03575* | PA14_53000 | *phhB* | Coenzyme | -1.55 | 3.46E-06 | Cytoplasmic |
| 17 | *00564* | PA14_63090 | *lldD* | Energy | -1.54 | 7.14E-05 | Cytoplasmic |
| 18 | *01624* | PA14_50470 | *flgC* | Cell motility | -1.51 | 5.07E-07 | Cytoplasmic |
| 19 | *01271* | PA14_45830 |  | Cell motility | -1.49 | 1.51E-03 | Extracellular |
| 20 | *03189* | PA14_57870 | *mlaE* | Secondary metabo | -1.43 | 4.55E-03 | Cytoplasmic |
| 21 | *01622* | PA14_50450 | *flgE* | Cell motility | -1.42 | 4.69E-05 | Unknown |
| 22 | *01625* | PA14_50480 | *flgB* | Cell motility | -1.40 | 3.21E-03 | IM |
| 23 | *02872* | PA14_38140 |  | Amino acid | -1.37 | 3.18E-03 | Cytoplasmic |
| 24 | *01604* | PA14_50200 | *fleS* | Signal transduc. | -1.30 | 7.11E-04 | IM |
| 25 | *01616* | PA14_50360 | *flgK* | Cell motility | -1.29 | 8.21E-06 | Cytoplasmic |
| 26 | *01269* | PA14_45800 | *fliM* | Cell motility | -1.28 | 6.15E-03 | IM |
| 27 | *03190* | PA14_57880 | *mlaF* | Secondary metabo | -1.21 | 1.02E-03 | IM |
| 28 | *00371* | PA14_65450 | *motA* | Cell motility | -1.17 | 4.61E-04 | Cytoplasmic |
| 29 | *05052* | PA14_20740 |  | Cell motility | -1.17 | 7.82E-03 | Cytoplasmic |
| 30 | *01597* | PA14_50080 | *fliJ* | IT/S/VT | -1.17 | 3.63E-03 | Cytoplasmic |
| 31 | *03210* | PA14_58080 |  | General function | -1.16 | 2.41E-03 | Cytoplasmic |
| 32 | *05291* | PA14_70280 | *argB* | Amino acid | -1.14 | 2.42E-04 | IM |
| 33 | *00477* | PA14_64180 |  | Translation, ribosomal structure and biogenesis | -1.12 | 3.18E-03 | Cytoplasmic |
| 34 | *01608* | PA14_50270 | *fliD* | Cell motility | -1.10 | 1.30E-03 | Cytoplasmic |
| 35 | *01260* | PA14_45680 | *flhA* | IT/S/VT | -1.06 | 2.75E-03 | Unknown |
| 36 | *00074* |  |  |  | -1.01 | 7.82E-03 | NA |
| 37 | *03960* | PA14_32630 |  | Secondary metabo. | 1.32 | 4.41E-03 | NA |
| **Rank** | **YIK_ID_IG**** | **PA14_ID_IG** | ***Name*** | **Predicted function*** | **Log_2_(FC)** | **P_adj_** | **Subcellular localization*** |
| 1 | 02678_IG |  | *pslD* | Envelope biogenesis | -4.14 | 2.45E-11 |  |
| 2 | 04792_IG | PA14_72010_IG |  | Envelope biogenesis | -2.52 | 4.46E-08 |  |

**Table S5. Full Tn-seq data in the strain YIK.**

**Table S6. Top hits of insertion mutants in PA14 genes enriched in plasma compared to heat inactivated plasma.**

**Predicted functions and subcellular localization based on homology with PAO1 genes* (3)

| Rank | **PA14_ID** | ***Name*** | **Predicted function*** | **Log_2_(FC)** | **P_adj_** | **Subcellular localization*** |
| --- | --- | --- | --- | --- | --- | --- |
| 1 | *PA14_64230* | *retS* | Signal transduction mechanisms | 18.66 | 8.88E-218 | IM |
| 2 | *PA14_52570* | *rsmA* | Signal transduction mechanisms | 17.24 | 2.18E-105 | Cytoplasmic |
| 3 | *PA14_64930* |  | Nucleotide transport and metabolism | 16.34 | 2.60E-117 | Unknown |
| 4 | *PA14_60650* |  | Function unknown | 16.02 | 7.84E-271 | Cytoplasmic |
| 5 | *PA14_43940* | *sucD* | Energy production and conversion | 15.32 | 4.19E-39 | Cytoplasmic |
| 6 | *PA14_28880* |  | Function unknown | 14.88 | 7.17E-40 | Cytoplasmic |
| 7 | *PA14_42400* | *exsB* | Function unknown | 14.43 | 6.46E-86 | OM |
| 8 | *PA14_46240* |  | Cell wall/membrane/envelope biogenesis | 14.05 | 5.76E-56 | IM |
| 9 | *PA14_16290* |  | Function unknown | 13.95 | 9.23E-40 | Cytoplasmic |
| 10 | *PA14_62580* | *panB* | Coenzyme transport and metabolism | 13.52 | 1.22E-29 | Unknown |
| 11 | *PA14_49700* |  | Transcription | 13.14 | 3.01E-30 | Cytoplasmic |
| 12 | *PA14_43950* | *sucC* | Energy production and conversion | 12.58 | 4.62E-39 | Cytoplasmic |
| 13 | *PA14_18780* |  | Defense mechanisms | 12.20 | 2.84E-89 | IM |
| 14 | *PA14_51880* | *oprD* | Function unknown | 12.11 | 1.73E-141 | OM |
| 15 | *PA14_69900* |  | Signal transduction mechanisms | 12.08 | 3.49E-124 | IM |
| 16 | *PA14_10550* |  | Inorganic ion transport and metabolism | 12.02 | 5.48E-85 | Cytoplasmic |
| 17 | *PA14_09750* |  | Amino acid transport and metabolism | 11.73 | 3.93E-211 | Cytoplasmic |
| 18 | *PA14_62590* | *panC* | Coenzyme transport and metabolism | 11.71 | 5.12E-40 | Cytoplasmic |
| 19 | *PA14_53590* |  |  | 11.58 | 9.54E-34 | NA |
| 20 | *PA14_17620* | *potC* | Amino acid transport and metabolism | 11.56 | 2.04E-60 | IM |
| 21 | *PA14_19960* |  | Function unknown | 11.50 | 4.32E-115 | Unknown |
| 22 | *PA14_26220* | *hisM* | Amino acid transport and metabolism | 11.43 | 0 | IM |
| 23 | *PA14_10410* |  |  | 11.01 | 2.97E-189 | NA |
| 24 | *PA14_43610* |  | Lipid transport and metabolism | 10.97 | 6.89E-66 | Cytoplasmic |
| 25 | *PA14_30970* |  |  | 10.71 | 6.87E-76 | NA |
| 26 | *PA14_19120* | *rhlR* | Transcription | 10.62 | 8.59E-250 | Cytoplasmic |
| 27 | *PA14_45000* | *gcl* | General function prediction only | 10.56 | 1.97E-61 | Cytoplasmic |
| 28 | *PA14_42600* | *pscP* | Function unknown | 10.56 | 2.43E-15 | Extracellular |
| 29 | *PA14_33770* | *pvdR* | Cell wall/membrane/envelope biogenesis | 10.50 | 5.37E-42 | IM |
| 30 | *PA14_30620* |  | Transcription | 10.44 | 6.45E-87 | Cytoplasmic |
| 31 | *PA14_48115* | *aprD* | General function prediction only | 10.43 | 7.65E-111 | IM |
| 32 | *PA14_01690* |  | General function prediction only | 10.41 | 1.95E-119 | IM |
| 33 | *PA14_15350* |  |  | 10.33 | 2.85E-62 | NA |
| 34 | *PA14_57500* |  | Carbohydrate transport and metabolism | 9.84 | 1.62E-11 | Cytoplasmic |
| 35 | *PA14_43460* |  | Lipid transport and metabolism | 9.70 | 4.25E-33 | Unknown |
| 36 | *PA14_64940* |  | Coenzyme transport and metabolism | 9.53 | 1.21E-60 | Cytoplasmic |
| 37 | *PA14_25430* |  | Cell wall/membrane/envelope biogenesis | 9.51 | 2.39E-09 | IM |
| 38 | *PA14_55480* |  | Intracellular trafficking, secretion, and vesicular transport | 9.49 | 3.22E-18 | IM |
| 39 | *PA14_28810* |  |  | 9.45 | 8.30E-114 | NA |
| 40 | *PA14_64110* | *accC* | Lipid transport and metabolism | 9.41 | 8.02E-16 | Cytoplasmic |

**Table S7. Top hits of insertion mutants in PA14 intergenic regions enriched in plasma compared to heat inactivated plasma.**

**Intergenic regions (IG) were assigned to the gene upstream independently of its orientation*

| **Rank** | **PA14_ID_IG*** | ***Name*** | **Predicted function**** | **Log_2_(FC)** | **P_adj_** |
| --- | --- | --- | --- | --- | --- |
| 1 | PA14_64950_IG |  | Secondary metabolites biosynthesis, transport and catabolism | 13.82 | 2.23E-33 |
| 2 | PA14_52580_IG | *lysC* | Amino acid transport and metabolism | 13.72 | 1.45E-20 |
| 3 | PA14_37980_IG |  | Signal transduction mechanisms | 11.97 | 8.69E-10 |
| 4 | PA14_52570_IG | *rsmA* | Signal transduction mechanisms | 11.72 | 9.63E-13 |
| 5 | PA14_05500_IG |  | Energy production and conversion | 11.57 | 2.70E-46 |
| 6 | PA14_64230_IG | *retS* | Signal transduction mechanisms | 11.09 | 2.48E-18 |
| 7 | PA14_64940_IG |  | Coenzyme transport and metabolism | 10.75 | 3.55E-27 |
| 8 | PA14_19320_IG |  | Function unknown | 10.44 | 6.98E-26 |
| 9 | PA14_53210_IG |  | Function unknown | 10.18 | 1.84E-50 |
| 10 | PA14_19350_IG |  | Carbohydrate transport and metabolism | 9.93 | 7.60E-20 |
| 11 | PA14_10420_IG | *tyrS* | Translation, ribosomal structure and biogenesis | 9.62 | 3.71E-19 |
| 12 | PA14_21030_IG |  | Intracellular trafficking, secretion, and vesicular transport | 9.17 | 2.74E-33 |
| 13 | PA14_34850_IG |  | Function unknown | 9.06 | 4.98E-10 |
| 14 | PA14_46240_IG |  | Cell wall/membrane/envelope biogenesis | 9.00 | 2.35E-05 |
| 15 | PA14_25180_IG | *psrA* | Transcription | 8.60 | 6.75E-06 |
| 16 | PA14_45680_IG | *flhA* | Intracellular trafficking, secretion, and vesicular transport | 8.31 | 1.41E-06 |
| 17 | PA14_03160_IG |  | Function unknown | 7.78 | 1.16E-07 |
| 18 | PA14_50530_IG | *braD* | Amino acid transport and metabolism | 7.61 | 6.77E-04 |
| 19 | PA14_52140_IG |  | Function unknown | 7.43 | 4.98E-10 |
| 20 | PA14_60860_IG | *nfxB* | Transcription | 7.41 | 1.11E-04 |
| 21 | PA14_57890_IG |  | General function prediction only | 7.36 | 9.12E-14 |
| 22 | PA14_41110_IG |  | Amino acid transport and metabolism | 7.36 | 1.87E-13 |
| 23 | PA14_41220_IG | *lon* | Posttranslational modification, protein turnover, chaperones | 7.24 | 2.76E-06 |
| 24 | PA14_29470_IG |  | Coenzyme transport and metabolism | 6.58 | 2.91E-03 |
| 25 | PA14_24440_IG |  | Function unknown | 6.55 | 5.89E-03 |
| 26 | PA14_41430_IG |  | Nucleotide transport and metabolism | 6.47 | 1.71E-03 |
| 27 | PA14_52310_IG | *dinB* | Replication, recombination and repair | 6.47 | 1.71E-03 |
| 28 | PA14_33510_IG |  | Function unknown | 6.43 | 1.93E-03 |
| 29 | PA14_45970_IG |  | Inorganic ion transport and metabolism | 6.24 | 7.24E-09 |
| 30 | PA14_09550_IG | *ppgL* | Carbohydrate transport and metabolism | 6.23 | 2.04E-03 |
| 31 | PA14_32420_IG |  | Energy production and conversion | 6.05 | 9.63E-13 |
| 32 | PA14_52990_IG | *phhA* | Amino acid transport and metabolism | 6.02 | 1.54E-03 |
| 33 | PA14_06720_IG | *nirF* | Function unknown | 5.96 | 1.17E-02 |
| 34 | PA14_53360_IG | *plcH* | Cell wall/membrane/envelope biogenesis | 5.79 | 9.21E-03 |
| 35 | PA14_64180_IG |  | Translation, ribosomal structure and biogenesis | 5.69 | 2.39E-04 |
| 36 | PA14_21050_IG |  | General function prediction only | 5.63 | 2.94E-03 |
| 37 | PA14_27675_IG |  |  | 5.61 | 2.05E-05 |
| 38 | PA14_22160_IG |  |  | 5.53 | 6.30E-08 |
| 39 | PA14_54850_IG |  |  | 5.53 | 1.48E-02 |
| 40 | PA14_30740_IG |  | General function prediction only | 5.52 | 2.39E-04 |

**Table S8. Full Tn-seq data in the strain PA14.**

**Table S9. Bacterial strains and plasmids**

| **Bacteria** | **Features / Source** | **Reference/origin** |
| --- | --- | --- |
| ***Pseudomonas aeruginosa*** |  |  |
| IHMA879472/AZPAE15042 | ExlA+, O11+O12, plasma sensitive / Urinary isolate | IHMA^1^ collection (4–6) |
| PA14 | T3SS+, ExoU+, O10/O19, plasma sensitive / Burn wound isolate | (6–8), Lory lab |
| CHA | T3SS+, ExoS+, O6, plasma resistant / Cystic fibrosis isolate | (9, 10) |
| YIK | T3SS+, ExoU+, O8, plasma resistant / Bacteremia | (6, 11) |
| CHA Δ*algR* | CHA with *algR* deletion | This work |
| CHA Δ*algD* | CHA with *algD* deletion | Lab collection |
| CHA Δ*crc* | CHA with *crc* deletion | This work |
| CHA Δ*pilQ* | CHA with *pilQ* deletion | This work |
| CHA Δ*wzy* | CHA with *wzy* deletion | This work |
| CHA Δ*wzz* | CHA with *wzz* deletion | This work |
| CHA Δ*ssg* | CHA with *ssg* deletion | This work |
| YIK Δ*flgC* | YIK with *flgC* deletion | This work |
| YIK Δ*gacS* | YIK with *gacS* deletion | This work |
| YIK Δ*pslE* | YIK with *pslE* deletion | This work |
| YIK Δ*ssg* | YIK with *ssg* deletion | This work |
| YIK Δ*crc* | YIK with *crc* deletion | This work |
| YIK Δ*crc* Δ*ssg* | YIK Δ*crc* with *ssg* deletion | This work |
| PA14 Tn::*retS* | PA14 with a transposon inserted in *retS* | This work |
|  |  |  |
| ***Escherichia coli*** |  |  |
| DH5α | Laboratory strain | Lab collection |
| TOP10 | Cloning strain | Invitrogen |
|  |  |  |
| **Plasmids** |  |  |
| pBTK24 | Plasmid with Himar-1 mariner transposon and C9 transposase (Amp^R^, Gm^R^) | (12) |
| pRK600 | Helper plasmid with conjugative properties (Cm^R^) | (13) |
| pEXG2 | Allelic exchange vector (Gm^R^), *sacB* | (14) |
| pEXG2-mut-*algR* | pEXG2 carrying DNA fragment for *algR* deletion in CHA obtained by SLIC (Gm^R^) | This work |
| pEXG2-mut-*crc* | pEXG2 carrying DNA fragment for *crc* deletion in CHA obtained by SLIC (Gm^R^) | This work |
| pEXG2-mut-*pilQ* | pEXG2 carrying DNA fragment for *pilQ* deletion in CHA obtained by SLIC (Gm^R^) | This work |
| pEXG2-mut-*wzy* | pEXG2 carrying DNA fragment for *wzy* deletion in CHA obtained by SLIC (Gm^R^) | This work |
| pEXG2-mut-*wzz* | pEXG2 carrying DNA fragment for *wzz* deletion in CHA obtained by SLIC (Gm^R^) | This work |
| pEXG2-mut-*ssg-CHA* | pEXG2 carrying DNA fragment for *ssg* deletion in CHA obtained by SLIC (Gm^R^) | This work |
| pEXG2-mut-*flgC* | pEXG2 carrying DNA fragment for *flgC* deletion in YIK obtained by SLIC (Gm^R^) | This work |
| pEXG2-mut-*gacS* | pEXG2 carrying DNA fragment for *gacS* deletion in YIK obtained by SLIC (Gm^R^) | This work |
| pEXG2-mut-*pslE* | pEXG2 carrying DNA fragment for *pslE* deletion in YIK obtained by SLIC (Gm^R^) | This work |
| pEXG2-mut-*ssg* | pEXG2 carrying DNA fragment for *ssg* deletion in YIK obtained by SLIC (Gm^R^) | This work |
| pEXG2-mut-*crc-YIK* | pEXG2 carrying DNA fragment for *crc* deletion in YIK obtained by SLIC (Gm^R^) | This work |

^1^ International Health Management Association, USA

**Table S10. Oligonucleotides used for PCRs**

| **Primers** | **Sequence (5’-3’)** |  |
| --- | --- | --- |
| pEXG2-mut-*algR*-sF1 | GGTCGACTCTAGAGGATCCCCTGGACCTGTCCGACCTGTTC | *algR* deletion in CHA |
| pEXG2-mut-*algR*-sR1 | CGCCAGAGGTTCGTCATCGA | *algR* deletion in CHA |
| pEXG2-mut-*algR*-sF2 | TCGATGACGAACCTCTGGCGTGAGGCGATGCGCTGACCGTCA | *algR* deletion in CHA |
| pEXG2-mut-*algR*-sR2 | ACCGAATTCGAGCTCGAGCCCCTGGCGTAGGTGTTCGAGAC | *algR* deletion in CHA |
| pEXG2-mut-*crc*-sF1 | GGTCGACTCTAGAGGATCCCCGCGGTGATCACGTCGTCGAT | *crc* deletion in CHA and YIK |
| pEXG2-mut-*crc*-sR1 | GGCCGCAGCCTGAATACCAT | *crc* deletion in CHA and YIK |
| pEXG2-mut-*crc*-sF2 | ATGGTATTCAGGCTGCGGCCTGACCGCTGATCGTCGACTACGA | *crc* deletion in CHA and YIK |
| pEXG2-mut-*crc*-sR2 | ACCGAATTCGAGCTCGAGCCCTGTTCGAACTTCAGCGCGCC | *crc* deletion in CHA and YIK |
| pEXG2-mut-*pilQ*-sF1 | GGTCGACTCTAGAGGATCCCCTGAGCATCCTGGCCAAGACC | *pilQ* deletion in CHA |
| pEXG2-mut-*pilQ*-sR1 | GCGCGAGAGGCCACTGTTC | *pilQ* deletion in CHA |
| pEXG2-mut-*pilQ*-sF2 | GAACAGTGGCCTCTCGCGCCAGGCCATCGCAATCGGTC | *pilQ* deletion in CHA |
| pEXG2-mut-*pilQ*-sR2 | ACCGAATTCGAGCTCGAGCCCATGCGGCGCGAACCAGCG | *pilQ* deletion in CHA |
| pEXG2-mut-*wzy*-sF1 | GGTCGACTCTAGAGGATCCCCCCAGCTTCCCTCGCCGCTT | *wzy* deletion in CHA |
| pEXG2-mut-*wzy*-sR1 | ACCGGTGAGCATCGCGTACA | *wzy* deletion in CHA |
| pEXG2-mut-*wzy*-sF2 | ATGTACGCGATGCTCACCGGTGAATCCGCCTTCCACAAGCTGG | *wzy* deletion in CHA |
| pEXG2-mut-*wzy*-sR2 | ACCGAATTCGAGCTCGAGCCCCCACTTATAAGCCCCGTCGG | *wzy* deletion in CHA |
| pEXG2-mut-*wzz*-sF1 | GGTCGACTCTAGAGGATCCCCACACTCCTGACGGTCCGCTT | *wzz* deletion in CHA |
| pEXG2-mut-*wzz*-sR1 | CAGATCGAACTCGCCGCTCT | *wzz* deletion in CHA |
| pEXG2-mut-*wzz*-sF2 | AGAGCGGCGAGTTCGATCTGACGGTCGATGCTGCATGAGT | *wzz* deletion in CHA |
| pEXG2-mut-*wzz*-sR2 | ACCGAATTCGAGCTCGAGCCCCCGGCGAAGAAGTCCTCGTT | *wzz* deletion in CHA |
| pEXG2-mut-*flgC*-sF1 | GGTCGACTCTAGAGGATCCCCGGTTCGCAGCCATGAGCATC | *flgC* deletion in YIK |
| pEXG2-mut-*flgC*-sR1 | CTCATGCCGCTACCGGCAAT | *flgC* deletion in YIK |
| pEXG2-mut-*flgC*-sF2 | ATTGCCGGTAGCGGCATGAGGCCTTCCAGACCAACGCGGA | *flgC* deletion in YIK |
| pEXG2-mut-*flgC*-sR2 | ACCGAATTCGAGCTCGAGCCCCGTTGCTGCTGGAGACCGG | *flgC* deletion in YIK |
| pEXG2-mut-*gacS*-sF1 | GGTCGACTCTAGAGGATCCCCCGGCGGAGAACTGCAGCGA | *gacS* deletion in YIK |
| pEXG2-mut-*gacS*-sR1 | GGTGAGCAGCAGTACGCGC | *gacS* deletion in YIK |
| pEXG2-mut-*gacS*-sF2 | GCGCGTACTGCTGCTCACCCTCTGACCATGCGCATCCTG | *gacS* deletion in YIK |
| pEXG2-mut-*gacS*-sR2 | ACCGAATTCGAGCTCGAGCCCCCACGGCCGGTATTGATCAG | *gacS* deletion in YIK |
| pEXG2-mut-*pslE*-sF1 | GGTCGACTCTAGAGGATCCCCAGCGTGCCCTGGAAGAACTC | *pslE* deletion in YIK |
| pEXG2-mut-*pslE*-sR1 | GCGCAACAGATCACGCAAGG | *pslE* deletion in YIK |
| pEXG2-mut-*pslE*-sF2 | CCTTGCGTGATCTGTTGCGCGGTCCTGCAGACCATCGCC | *pslE* deletion in YIK |
| pEXG2-mut-*pslE*-sR2 | ACCGAATTCGAGCTCGAGCCCGGTCGAGGTAGTTGCCGCC | *pslE* deletion in YIK |
| pEXG2-mut-*ssg*-sF1 | GGTCGACTCTAGAGGATCCCCTCTGGACTGGCAGCCGCAG | *ssg* deletion in YIK and CHA |
| pEXG2-mut-*ssg*-sR1 | ATCCAGGATCGCGCGCTGC | *ssg* deletion in YIK and CHA |
| pEXG2-mut-*ssg*-sF2 | GCAGCGCGCGATCCTGGATTGAGATGGCTGGAGAAGACCTGG | *ssg* deletion in YIK and CHA |
| pEXG2-mut-*ssg*-sR2 | ACCGAATTCGAGCTCGAGCCCTCCAGGCGTTGCGGCAACAT | *ssg* deletion in YIK and CHA |
|  |  |  |
| **Primers for Tn-seq** |  |  |
| Short adpator | TACCACGACCA-NH2 |  |
| Long adaptor | GTGACTGGAGTTCAGACGTGTGCTCTTCCGATCTGGTCGTGGTAT |  |
| PCR1 Tn-specific | CACAGGAAACAGGACTCTAGAGG |  |
| PCR2 adaptor complementary | GTGACTGGAGTTCAGACGTGTG |  |
| P5+ Illumina | AATGATACGGCGACCACCGAGATCTACACTCTTTCCCTACACGACGCTCTTCCGATCTCTAGAGACCGGGGACTTATCAGC |  |
| P7-index | CAAGCAGAAGACGGCATACGAGATNNNNNN |  |

**References**

1. Janet-Maitre M, Pont S, Masson FM, Sleiman S, Trouillon J, Robert-Genthon M, Gallet B, Dumestre-Perard C, Elsen S, Moriscot C, Bardoel BW, Rooijakkers SHM, Cretin F, Attrée I. 2023. Genome-wide screen in human plasma identifies multifaceted complement evasion of Pseudomonas aeruginosa. PLoS Pathog 19:e1011023.

2. Cock PJA, Chilton JM, Grüning B, Johnson JE, Soranzo N. 2015. NCBI BLAST+ integrated into Galaxy. GigaScience 4:s13742-015-0080–7.

3. Winsor GL, Griffiths EJ, Lo R, Dhillon BK, Shay JA, Brinkman FSL. 2016. Enhanced annotations and features for comparing thousands of Pseudomonas genomes in the Pseudomonas genome database. Nucleic Acids Research 44:D646–D653.

4. Kos VN, Deraspe M, McLaughlin RE, Whiteaker JD, Roy PH, Alm RA, Corbeil J, Gardner H. 2015. The resistome of Pseudomonas aeruginosa in relationship to phenotypic susceptibility. Antimicrobial agents and chemotherapy 59:427–36.

5. Trouillon J, Sentausa E, Ragno M, Robert-Genthon M, Lory S, Attree I, Elsen S. 2020. Species-specific recruitment of transcription factors dictates toxin expression. Nucleic acids research 48:2388–2400.

6. Pont S, Fraikin N, Caspar Y, Van Melderen L, Attree I, Cretin F. 2020. Bacterial behavior in human blood reveals complement evaders with some persister-like features. PLoS pathogens 16:e1008893.

7. Rahme LG, Stevens EJ, Wolfort SF, Shao J, Tompkins RG, Ausubel FM. 1995. Common virulence factors for bacterial pathogenicity in plants and animals. Science 268:1899–1902.

8. Hao Y, Murphy K, Lo RY, Khursigara CM, Lam JS. 2015. Single-Nucleotide Polymorphisms Found in the migA and wbpX Glycosyltransferase Genes Account for the Intrinsic Lipopolysaccharide Defects Exhibited by Pseudomonas aeruginosa PA14. J Bacteriol 197:2780–2791.

9. Toussaint B, Delicattree I, Vignais PM. 1993. Pseudomonas aeruginosa Contains an IHF-like Protein That Binds to the algD Promoter. Biochemical and Biophysical Research Communications 196:416–421.

10. Bezuidt OK, Klockgether J, Elsen S, Attree I, Davenport CF, Tümmler B. 2013. Intraclonal genome diversity of Pseudomonas aeruginosa clones CHA and TB. BMC Genomics 14:416.

11. Elabbadi A, Pont S, Verdet C, Plésiat P, Cretin F, Voiriot G, Fartoukh M, Djibré M. 2020. An unusual community-acquired invasive and multi systemic infection due to ExoU-harboring Pseudomonas aeruginosa strain: Clinical disease and microbiological characteristics. Journal of Microbiology, Immunology and Infection 53:647–651.

12. Kulasekara HD, Ventre I, Kulasekara BR, Lazdunski A, Filloux A, Lory S. 2005. A novel two-component system controls the expression of Pseudomonas aeruginosa fimbrial cup genes. Molecular Microbiology 55:368–380.

13. Kessler B, de Lorenzo V, Timmis KN. 1992. A general system to integratelacZ fusions into the chromosomes of gram-negative eubacteria: regulation of thePm promoter of theTOL plasmid studied with all controlling elements in monocopy. Molecular and General Genetics MGG 233:293–301.

14. Rietsch A, Vallet-Gely I, Dove SL, Mekalanos JJ. 2005. ExsE, a secreted regulator of type III secretion genes in Pseudomonas aeruginosa. PNAS 102:8006–8011.
